# The Electrosolvation Effect: The Medium Is the Message

**DOI:** 10.1021/acs.langmuir.6c02385

**Published:** 2026-09-09

**Authors:** Sida Wang, Ali Behjatian, Angela Le, Xin Zhu, Madhavi Krishnan

**Affiliations:** † Physical and Theoretical Chemistry Laboratory, Department of Chemistry, 6396 University of Oxford, South Parks Road, Oxford OX1 3QZ, United Kingdom; ‡ The Kavli Institute for Nanoscience Discovery, Sherrington Road, Oxford OX1 3QU, United Kingdom

## Abstract

It is a well-known principle that like charges repel, whether positive or negative. However, experiments on electrically charged matter suspended in liquids have frequently reported the opposite: attraction between like-charged particles, rather than repulsion. Experiments have also shown that the sign of the force at large separations can depend on the sign of the particle charge, e.g., in water negative particles attract, while positives repel as expected, and this trend generally reverses in polar organic solvents such as alcohols. These observations cannot be reconciled with standing theories of the electrostatic interaction that regard the intervening solvent medium as a structureless, featureless dielectric continuum. Recent work has called for the traditional continuum view of the medium to be relinquished, attributing the observed attraction to an “electrosolvation force” – a term that describes interactions bearing electrical signatures that depend on the properties and structuring of the molecular solvent close to an interface. Here we discuss a range of recent experiments that shed light on the mechanistic underpinnings of this previously unanticipated interaction. We also discuss developments on the theoretical front that not only capture the observed attraction between like-charged particles but also recover the canonical monotonic repulsion – a hallmark of standing continuum-dielectric theories. Thus, the traditionally anticipated repulsion represents a distinct and important regime within a general theoretical framework that accounts for the solvent in substantially greater detail. Given the fundamental importance of the forces that drive structure and organization from molecular and particulate building blocks, this emerging discovery is likely to have broad implications across a wide range of fields. Interface-governed properties that generate long-range signatures in fluids may also prove relevant to electrokinetic and electrochemical processes.

## Graphical abstract

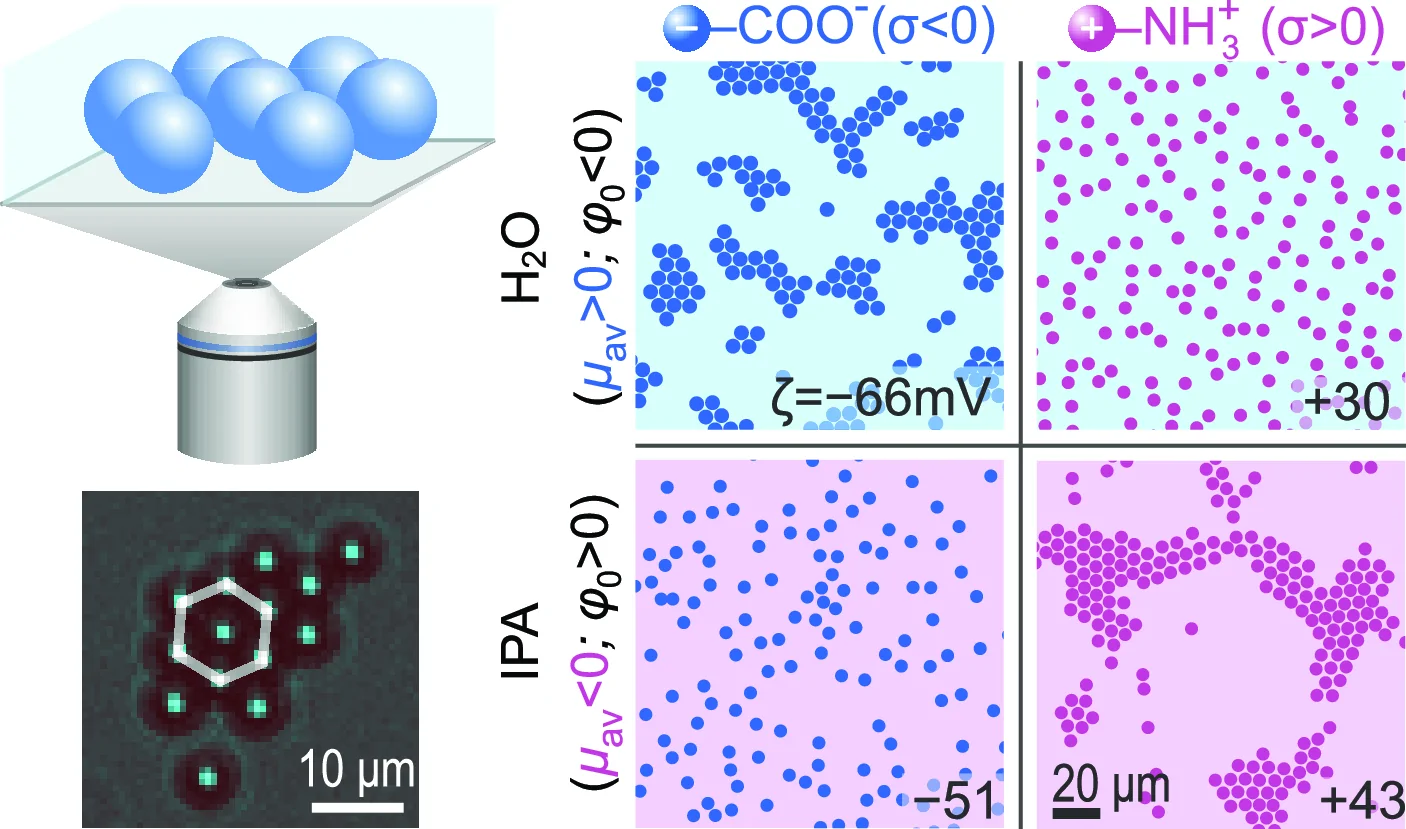

## Introduction

Surfaces and interfaces play a critical role in the behavior of macroscopic systems. The solid-liquid interface in water in particular underpins a host of phenomena encountered in geology, the atmosphere, biology, and in technological applications such as electrochemistry, energy production and storage devices, biological implants and drug design.^1,2^ The importance of understanding the physics and chemistry of interfacial processes and the way they impact natural and technological processes therefore cannot be overstated. This review summarizes recent developments in the understanding of the role that interfaces play in the interactions between particles in the fluid phase, with a specific focus on electrically charged matter.

An interface as the name connotes does not exist in isolation, but is intimately associated with a bulk medium. Given the central role of interfacial phenomena across many contexts, the possibility that interfaces and their surrounding media may couple to produce emergent behavior in the bulk phase warrants close scrutiny. Yet, with a few important exceptions, our view of interparticle interactions in the solution phase largely continues to mirror Barry Ninham’s assessment in the 1980s: “We tend to regard an intervening medium as an annoying complication and by a process of sympathetic magic sweep the whole mess into an effective vacuum or uniform continuum wherein, in truth, sits all the interesting physics. . .. Because of the successes of the DLVO theory, we do know, or think that we know, that in dealing with attractive and repulsive forces between colloidal particles, it is *usually* sufficient to replace the solvent by a continuum··· *But we are not completely sure*.”.^3^ The present review is, in large measure, a tribute to this observation. We focus on a central, rather general, yet highly counterintuitive phenomenon in interparticle interactions in the solution phase that contradicts the predictions of standing continuum theories. In particular we discuss the recently introduced view that the interfacial electrolyte plays a pivotal role in triggering long-ranged forces between objects immersed in a fluid.^4–9^

We recently coined the term “electrosolvation” to describe charge-coupled or charge-driven solvation effects that can be attributed to the properties of solvent molecules at the solid-liquid interface.^8^ Our observations indicate that the spatial properties and structuring of solvent molecules at an interface play a defining role in launching and sustaining forces between objects in solution. It appears, then, that a substantial body of prior observations and an expanding base of experimental evidence may be more fully understood by accounting for the intricate—and often overlooked—role of the interfacial solvent. Our goal is to highlight a range of experimental features characterizing this newly introduced principle in interactions, raising the possibility of its relevance in a range of fields in Chemistry and Biology where interactions in the fluid phase play a foundational role, e.g., in biological organization problems such as chromatin condensation, liquid-liquid phase segregation, protein crystallization, multimerization, aggregation, coagulation, and even in biomolecular folding and chemical reactivity.

Life is built on the interaction of biomolecular species – DNA, proteins and lipids – all of which carry significant numbers of electrically charged groups. A growing body of evidence has indicated that our current picture of interactions in charged matter in solution may be due for significant revision. Indeed over the past several decades, the literature has consistently reported observations of a long-ranged attraction between objects in solution that carried electrical charge of the same sign. Experimental evidence for this counterintuitive attraction and discussions of its origins may in fact date as far back as a century,^10^ including an analysis by Langmuir on “unipolar coacervation” in the 1930s, and the observations of Rosalind Franklin et al. on crystallization of viruses later in the 1950s.^11,12^ In a completely different context, several pioneering reports on the crystal structure of RNA reveal the surprising presence of parallel or nearly parallel arrangements of helices.^13,14^ In fact parallel packing is observed in more than 50% of all packing interactions in crystals of A-form RNA, in conflict with the intuitively expected strong charge repulsion between the helices, and has not found a satisfactory physical explanation to date.^14^ Moreover there exists a substantial body of literature on the ability of DNA to self-attract even in the absence of divalent cations and proteins, which carries obvious and important implications for fundamental biological processes such as chromatin organization and homologous recombination.^14–20^

Since the early reports by Klug, Franklin and co-workers on virus clustering, observations of counterintuitive attraction between similarly charged particles have posed a direct challenge to our understanding of interparticle and intermolecular interactions in the fluid phase, that are expected to be described by the Derjaguin–Landau–Verwey–Overbeek (DLVO) theory.^21–23^ We note parenthetically that viruses generally carry an overall net negative charge at physiological pH. The 1990s saw a re-emergence of interest in the notion of an attractive force between electrically similarly charged particles in aqueous solution, that was likely to be the same force alluded to in the early work on viruses. The epithet ‘like-charge attraction’ was coined, referring to the family of measurements on a rather weak and long ranged attraction whose strength was of the order of 0.5 *k*_B_ *T* between micron-sized spheres in solution.^24–26^ Numerous theoretical studies examined the problem, and some that initially appeared to resolve it ultimately proved to be flawed.^27–30^ The investigation eventually became mired in concerns around potential artifacts in the experimental data, leaving the overall effort largely inconclusive in the decades that followed.^31^ The phenomenon as such continued to pose an unresolved and open question until the advent of rather different families of experimental observations which reinvigorated interest in the question.^32–35^

We recently proposed a theoretical view of an electrosolvation force that arises on account of the solvent itself and could explain the anomalous attraction.^4^ The suggested interaction mechanism is founded in the properties of interfacial solvent molecules, similar to the well-known short-range hydration force. However, the two interactions are rather different in the broadest sense. Unlike the hydration force the electrosolvation force (1) is subject to explicit electrical control, in that its sign and magnitude can be modulated by that of the electrical charge on the interacting objects, and (2) operates even at long-range (separations of ca. 10 κ^–1^ or greater) between objects in solution.^4,5,7–9^ Here κ^–1^ denotes the familiar Debye length which represents the spatial rate of decay of electrical fields in the medium, and may be approximated by the value 0.3/√c nanometers in an aqueous electrolyte containing monovalent salt at a molar concentration *c*.

### Relationship to the Long-Range Hydrophobic Attraction and Other “Non-DLVO” Forces

Over the past few decades, measurements of forces between nominally charge-neutral hydrophobic surfaces interacting in water have revealed a “long-range hydrophobic attraction”. These forces have a range of 10s, if not 100s of nanometers – far longer than the canonical correlation lengths in water (1–3 Å) and are worth considering briefly in the present context.^36–40^ In our view, the electrosolvation effect is likely to provide a general overarching mechanism of which a number of other apparently anomalous phenomena may prove to be special cases. For example, “like-charge attraction” may be regarded as one fascinating consequence of the electrosolvation mechanism. But electrosolvation effects may in fact drive more broad and general results such as mutual repulsion, and indeed attraction – the hydrophobic attraction – between formally uncharged or near charge-neutral surfaces. Thus, the sense in which we generally use the term “electrosolvation” refers to solvation governed phenomena that give rise to forces with clear electrical signatures when they act between charge carrying objects. But the same core mechanism may be expected to explain forces between formally charge-neutral objects, as in fact previously alluded to in ref 41 on the long-range hydrophobic force.

It is also important to emphasize at the outset, that although electrosolvation forces are expected to act rather generally, this does not guarantee the observation of a *measurable attraction* that outweighs the canonical repulsion between similarly charged particles. In fact, both the experiments and proposed theoretical model(s) of the interaction clearly show that observation of cluster formation requires specific parametric conditions to be met in experiment, and that attraction should therefore not be expected as the norm. Indeed the canonically expected “DLVO-like” monotonically repulsive behavior between like-charged particles is often observed in experiment, and is naturally obtained within the conceptual framework of electrosolvation (see Theory Section). However, the converse does not hold: the DLVO view does not envisage attraction between like-charged particles. This is because the Poisson–Boltzmann theory, on which the DLVO model is based, does not offer any room for (1) a long-range attraction between like charged particles in a dilute aqueous electrolyte,^29,30^ or (2) the breaking of “sign-reversal symmetry” in the observed self-attraction (i.e., in general, depending on the solvent, either positive or negative particles are observed to attract in experiments). The problem under consideration is also fundamentally different from a class of “non-DLVO” attractive forces that arise when electrostatic considerations situate the problem decidedly outside the remit of PB theory, e.g., in the strong coupling regime of low dielectric constant, multivalent ions, and high salt concentration.^42,43^

Although the electrosolvation mechanism originates from the structure and orientation of solvent molecules within what is commonly referred to as the Stern layer, the longer-ranged consequences of this interfacial solvent organization—and in particular its role in mediating interactions between objects in solution—have not, to our knowledge, been previously recognized. The evidence thus points to the electrosolvation force as a fundamentally new class of interaction that emerges from interfacial solvent organization. Moreover, in our present understanding of electrosolvation forces, solvent orientation at an interface – as captured by solvent polarization – can influence the nature of ion distributions in the vicinity.^44,45^ Therefore, it is possible that electrosolvation effects also play a role in electrochemical processes involving, e.g., electrokinetic mobility, as well as ion selectivity of interfaces and confined fluid-filled geometries in fluids.^46^ Our current theoretical perspective on electrosolvation effects is described further in the Theory Section of this paper. A later section of the manuscript discusses possible links between interfacial processes governing interactions and their implications for electrochemically relevant observables and phenomena.

## Inferring Interparticle Interactions from Imaging Suspensions of Colloidal Microspheres in 2D

Experimental investigation of the interaction between charged bodies in the solution-phase has historically relied on predominantly mechanical approaches to measuring forces between macroscopic surfaces, e.g., Surface Force Apparatus (SFA) and Atomic Force Microscopy (AFM).^47,48^ These approaches have shed vital and much needed light on fundamental aspects of the interaction, but mechanical approaches are generally not sensitive to the weak forces at play in the case of small objects such as particles and molecules in solution. E.g., the scale of the forces of interest in the interparticle attraction in refs 7,9 corresponds to force per unit radius values of about 10 nN/m at most, which is 2–3 orders of magnitude smaller than the force sensitivity of typical SFA measurements. Measuring weak interactions in the sub-pN to fN force regime would require experimental techniques with much higher force sensitivity. Indeed optical microscopy-based measurement of interactions has long been recognized as delivering orders of magnitude better force sensitivity than mechanical approaches,^49^ and has also proven highly fruitful in our own measurements of weak interactions of nanoparticles and molecules in solution.^50–56^ Perhaps unsurprisingly, evidence for attraction between like-charged particles in fluids has overwhelmingly been obtained using radiation-based techniques, including X-ray, neutron, and light scattering, and optical microscopy, which infer interparticle interactions indirectly from the structure of suspensions.^11,12,24,57–61^

Here we focus on recent experiments that measure colloidal interactions using optical microscopy. The experimental system for the observation of colloidal cluster formation is relatively simple and is inspired by experiments originally described in ref 32. In a typical experiment, microspheres are rinsed several times by centrifugation and resuspension in the solvent or electrolyte of interest. Experiments generally involve no added salt to the electrolyte, with a nominal ion concentration of *c* ≈ 10^–5^ M in experiments in aqueous media. A small volume of the particle suspension is placed in a microscopy compatible quartz cell, and particles are allowed to sediment to the bottom of the cell (Figure 1A). Repulsion between the negatively charged quartz surface and negatively charged microspheres ensures that the particles levitate at a well-defined height above the surface corresponding to an intersurface separation of about 0.5 μm based on measurements reported for a similar system in ref 62. In experiments with positively charged particles, we coat the microscopy coverglass with a positively charged polymer – typically a cationic polyelectrolyte, in order to ensure repulsion and levitation of particles above the surface.^7,8^ This experimental setup provides an effectively two-dimensional suspension of mobile particles in which colloidal interactions can be directly observed and accurately quantified. Note that the presence of the underlying microscopy cover glass surface cannot be eliminated in the current measurement format. However, extensive investigations of cluster formation above glass surfaces coated with various polymers, as well as with both spatially patterned and uniform metal films, have demonstrated that the properties of this surface have a negligible influence on the interparticle interaction^7^ (see Section S4.4 of ref 7). This key feature of our experiments contrasts with earlier reports that confinement and/or the presence of charged walls in the vicinity were necessary for like-charge attraction to occur.^26,63^ Rather, our observations align with reports of interparticle attraction observed and measured for submicrometer scale anionic matter, generally in bulk solution.^11,12,57–61,64^ Also, in our work, degassing of the particle suspensions does not alter the observations.

**Figure 1:**
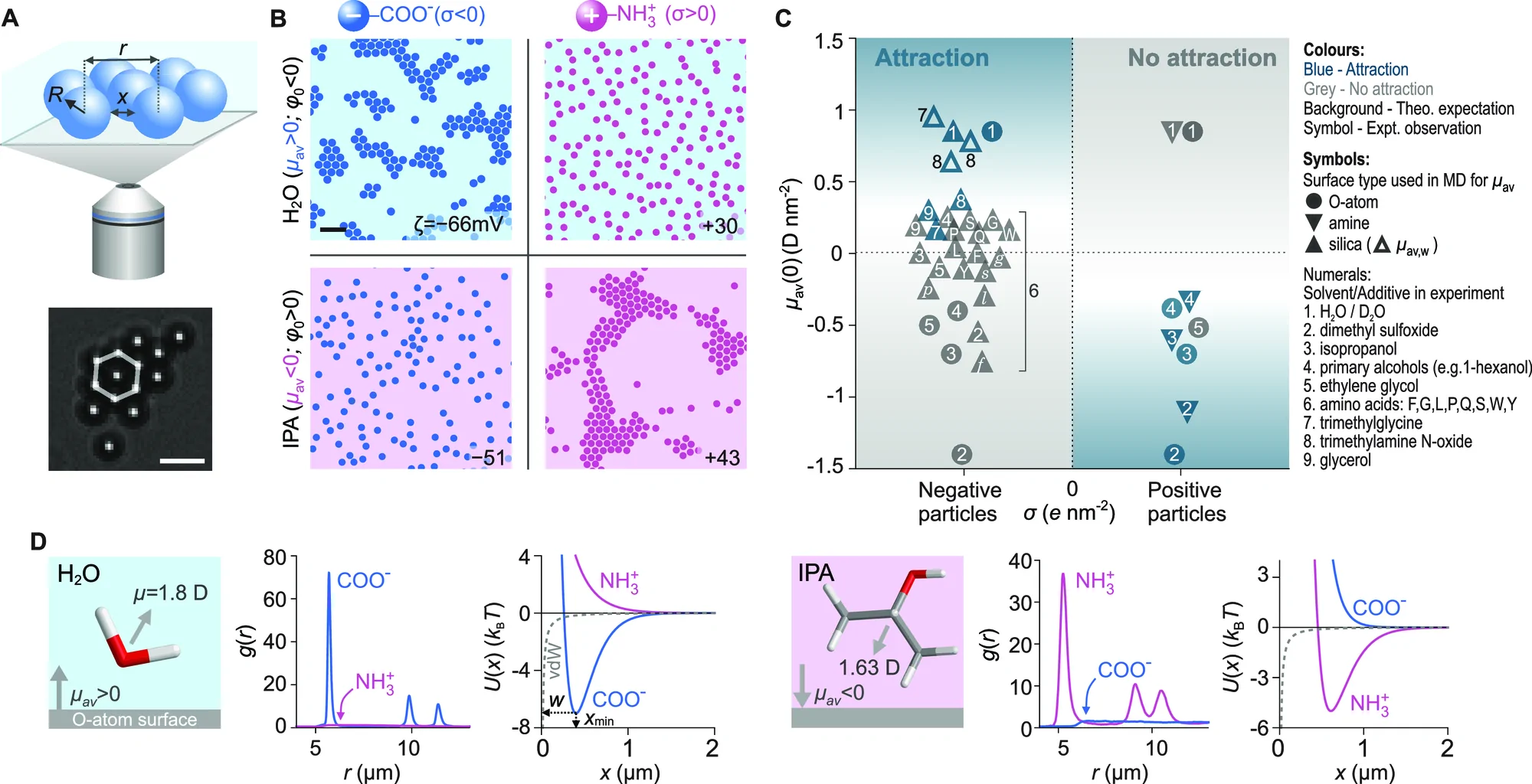
**Conceptual overview of the charge-asymmetric electrosolvation force. A** Optical microscopy setup illustrating carboxyl (COO^–^) functionalized particles of radius *R* attracting in water and forming clusters where the intersurface separation is *x*, and the center to center separation is *r* (top). Microscopy image of a representative particle cluster (bottom). Scale bar 10 μm. **B** Summary of the solvent and charge dependence of the electrosolvation force. Digitised microscopy images of microspheres ca. 5 μm in diameter that are positively charged (pink) and negatively charged (blue) suspended either in water (blue background) or in isopropanol (IPA) (pink background). Negatively charged carboxyl functionalized particles in water and positively charged aminated silica particles (NH_3_^+^) in IPA form clusters driven by a long-ranged interparticle attraction. Positively charged particles in water and negatively charged particles in IPA repel as intuitively expected. Measured zeta potentials are noted. Scale bar 20 μm. **C** Graphical summary of approximately 40 experiments exploring cluster formation under various conditions in the (μ_av_,σ) parameter space. μ_av_ and σ represent the normal component of the net interfacial dipole moment density and the surface charge density of the particle, respectively. Symbols denote experimental conditions, and colored background shading in the four quadrants indicates the theoretical expectation. Blue and gray colors denote attraction and cluster formation, and absence of attraction, respectively. **D** Schematic representation of the average molecular orientation of water and IPA at an uncharged oxygen-atom surface (molecular dipole moments μ – gray arrows) as inferred from MD simulations. μ_av_ points slightly away from the surface for H_2_O and toward the surface for IPA (dark gray arrows, left panels). Measured radial distribution functions *g*(*r*) of the 2D particle distributions display the periodic peak-structure characteristic of suspensions of ordered clusters, while the *g*(*r*) profiles for the two cases with no evident attraction are relatively featureless (middle panels). Pair interaction potentials inferred by matching *g*(*r*) profiles from BD simulations to experimental profiles (solid curves), and van der Waals (vdW) contribution to the total interaction (dashed curve) (right panels). *w* and *x*_min_ denote the depth and location of the inferred potential minimum, respectively. Adapted from refs 7, 8 . Available under a CC-BY 4.0 license. Copyright 2024 Wang, S., Walker-Gibbons, R., Watkins, B., Flynn, M., Krishnan, M and Copyright 2025 Wang, S., Walker-Gibbons, R., Watkins, B., Lin, B., Krishnan, M., respectively.

Working with colloidal microspheres we have demonstrated that a long-ranged attraction can drive the formation of ordered, slowly reorganizing clusters in solution, also referred to as “flocs” in traditional colloid science terminology (Figure 1A, B). Note that unlike the traditional view, flocculation here is driven by a force far stronger and more long ranged than that due to the van der Waals interaction^7,9^ (Figure 1D). The force can be tuned using a number of experimental knobs, such as the nature of the solvent, the p*H* and salt concentration in solution and surface chemistry. Unsurprisingly, surface active agents such as amino acids that are known to adsorb to surfaces, and surfactants such as Tween20, abolish attractions when present at very low concentrations in solution (Figure 1C). All these experiments exhibit features that point to the importance of the interfacial solvent structure in generating the observed long-range interparticle force. The experiments performed to date, and reviewed here, cover a few ways in which the long-range interaction may be tuned and controlled, with further avenues to modulating the interaction likely to be identified in future.^8^

## The Role of Solvents and pH in Determining the Magnitude of the Attraction

Figure 1B presents a graphical summary of the charge-asymmetric attraction driving cluster formation in particles in suspension. Unmodified silica particles are negatively charged in both water and heavy water, and can, depending on the p*H*, form ordered slowly reorganizing clusters indicative of a significant attractive force between the particles. Their positively charged, aminated counterparts however repel as intuitively expected. Suspending particles in solvents such as alcohols and dimethyl sulfoxide (DMSO) inverts the effect: now positively charged particles may attract and form clusters, while negative particles repel (Figure 2).

**Figure 2:**
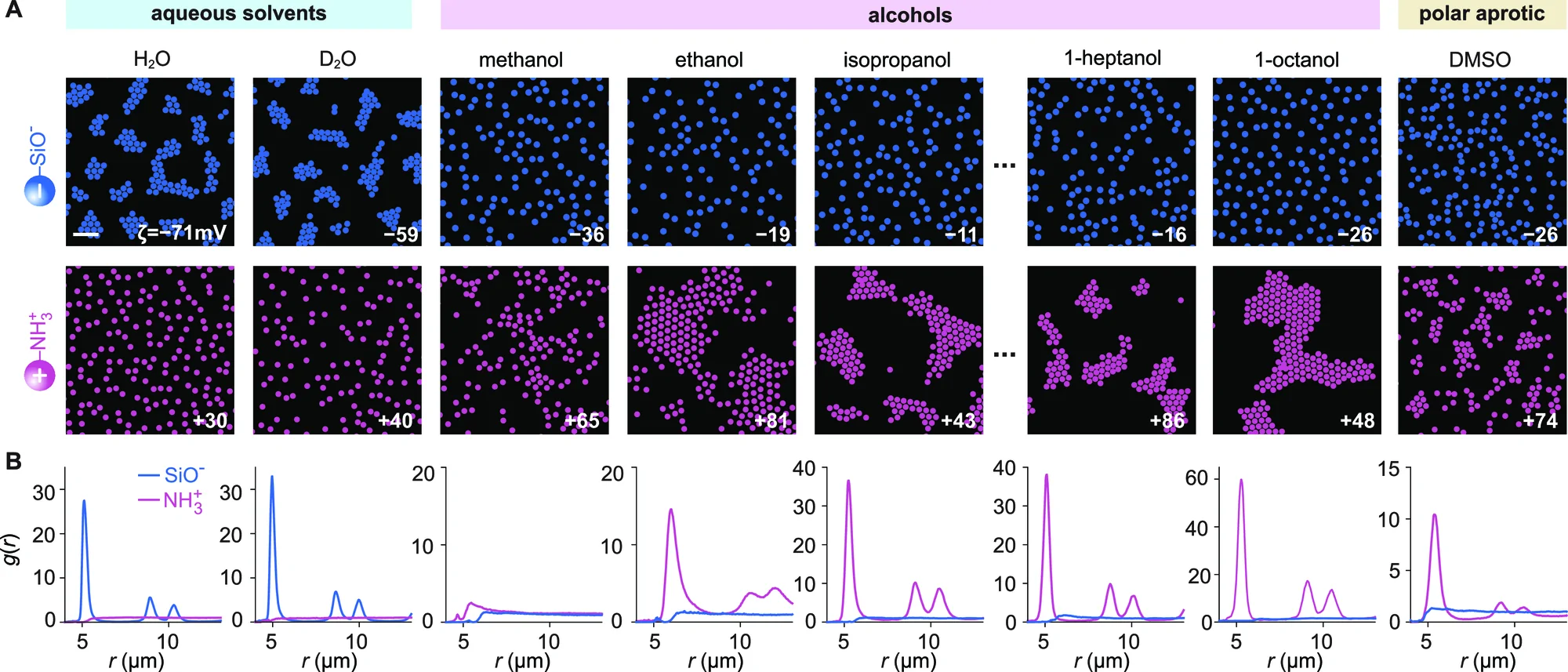
**Solvent dependence of charge-asymmetric cluster formation. A** Digitised microscopy images of two-dimensional colloidal suspensions of ca. 5 μm diameter SiO_2_ particles (blue) and positively charged aminated silica microspheres (pink) suspended in aqueous media, in alcohols with different chain lengths and in dimethyl sulfoxide (DMSO), a polar aprotic solvent (left to right). Clusters were observed in H_2_O and D_2_O for negatively charged SiO_2_ particles, and in alcohols and DMSO for positively charged aminated particles. Measured zeta potentials, ζ (in mV) are noted. Scale bar: 20 μm. **B** Measured *g*(*r*) profiles for negatively charged (blue lines) and positively charged particles (pink lines). Adapted from ref 8. Available under a CC-BY 4.0 license. Copyright 2025 Wang, S., Walker-Gibbons, R., Watkins, B., Lin, B., Krishnan, M.

We attribute the attraction, and the breaking of charge-symmetry in the interaction, to “charge-asymmetric solvation”. It was recognized early in the context of ion solvation that solvation free energies for cations and anions are solvent-dependent, and that the solvation structure around cations and anions is not only qualitatively and quantitatively different but is also solvent dependent.^65–68^ More recently, interfacial spectroscopy measurements have reported differences in the solvation structure with respect to the sign of surface charge at macroscopic interfaces.^69–71^ To explain the experimental observations on interparticle interactions we invoked a specific facet of interfacial solvation arising from the inherent molecular asymmetry of the water molecule.^4^ An interface in a fluid breaks the isotropy of bonding interactions for interfacial molecules in the fluid half-space (Figure 1D). As a result, even at an electrically neutral surface immersed in a fluid, solvent dipoles possess a tiny yet non-negligible amount of net orientation or *excess* polarization, which has been reported on extensively in computational work and spectroscopy on aqueous interfaces^4,72–76^ (molecular schematics in Figure 1D). Naturally, this net orientation is also influenced by electrical charging of the surface. Overall, the presence of net solvent orientation at a neutral surface breaks the symmetry in solvation free energy with respect to the sign of charge on the surface.^4^ In an early version of our theoretical model we parametrized the excess polarization in terms of an interfacial solvation potential φ_0_ for an uncharged surface, or φ_int_ for a charged surface (Figure 1B).^4,5,7^ The parameter φ_0_ in our original model is analogous to the χ-potential in electrochemistry.^77^

We determined the free energy change due to this prepolarized layer of interfacial solvent molecules for two approaching particles capable of undergoing charge regulation.^4^ Adding this free energy to the traditional (PB) electrostatic free energy of repulsion between like charged particles, we were able to qualitatively explain many of the experimentally observed features of the interparticle interaction, as described further in the Theory Section.^4^ The orientation of water at an interface is such that negative particles are expected to attract in water and heavy water, and positive particles to repel, i.e., in these solvents the net normal dipole moment density μ_av_ > 0 (φ_0_ < 0). In other solvents such as alcohols, MD simulations suggested that net normal dipole moment due to solvent orientation at an interface was inverted compared to water (μ_av_ < 0,φ_0_ > 0) (Figure 1C). Based on the interfacial solvation model, this indicated that positive particles should attract in experiment and negative particles should repel. Experiments conducted in a range of different solvents indeed largely confirmed the correlation between the sign of polarization expected to develop at a neutral surface immersed in that solvent and the observation that either negative or positive particles attracted in that medium (Figure 2). In fact, over 40 experimental conditions tested to date conform to the overall expectation of attraction or repulsion based on the interfacial solvation model (Figure 1C).

However, experiments performed in solvents such as ethylene glycol (EG), which has two structural conformers with vastly different dipole moments, displayed interesting departures from the simple expectation (Figure 3A). MD simulations of model oxygen-atom surfaces immersed in EG suggested that the sign of the net normal solvent dipole moment density at a neutral oxygen atom surface is negative (μ_av_ < 0), indicating that positively charged particles should attract, provided that the orientational ordering of solvent molecules at the interface were strong enough. μ_av_ < 0 further implies that negatively charged particles should repel. Experiments however revealed no attraction for positively charged aminated silica particles in EG, even over a wide range of p*H* altered by adding acid and base to the medium. Negatively charged particles displayed a repulsion rather than an attraction, which is qualitatively expected within the proposed theoretical view. The observations on EG point to the importance of and challenges associated with accurately simulating solvents at interfaces in a manner that correctly accounts for the effects of specific surface chemistry. For instance, when we refer to the influence of “positively and negatively charged” particles on the interfacial solvent, what we contend with in practice is the solvation of carboxyl, silanol, sulfonate and phosphate groups for negatively charged systems, and amine groups and quaternary ammonium cations for positively charged objects. The influence of surface group chemistry on solvation can hardly be captured by studying solvation of a 2d assembly of charge-carrying oxygen atoms used in simulations. In fact MD simulations of water at silanol surfaces with varying hydroxyl group densities show that the bonding interactions of the surface ionizable groups significantly influence the structure of the interfacial solvent.^6,8^ Our MD simulations of solvent interfaces have however largely relied on an assembly of oxygen-atoms to provide a simple description of a surface. Thus, important aspects of the chemical influence of the interfacial groups on solvation structure are inevitably overlooked within a simplistic binary, sign-dominated view of interfacial solvation.

**Figure 3:**
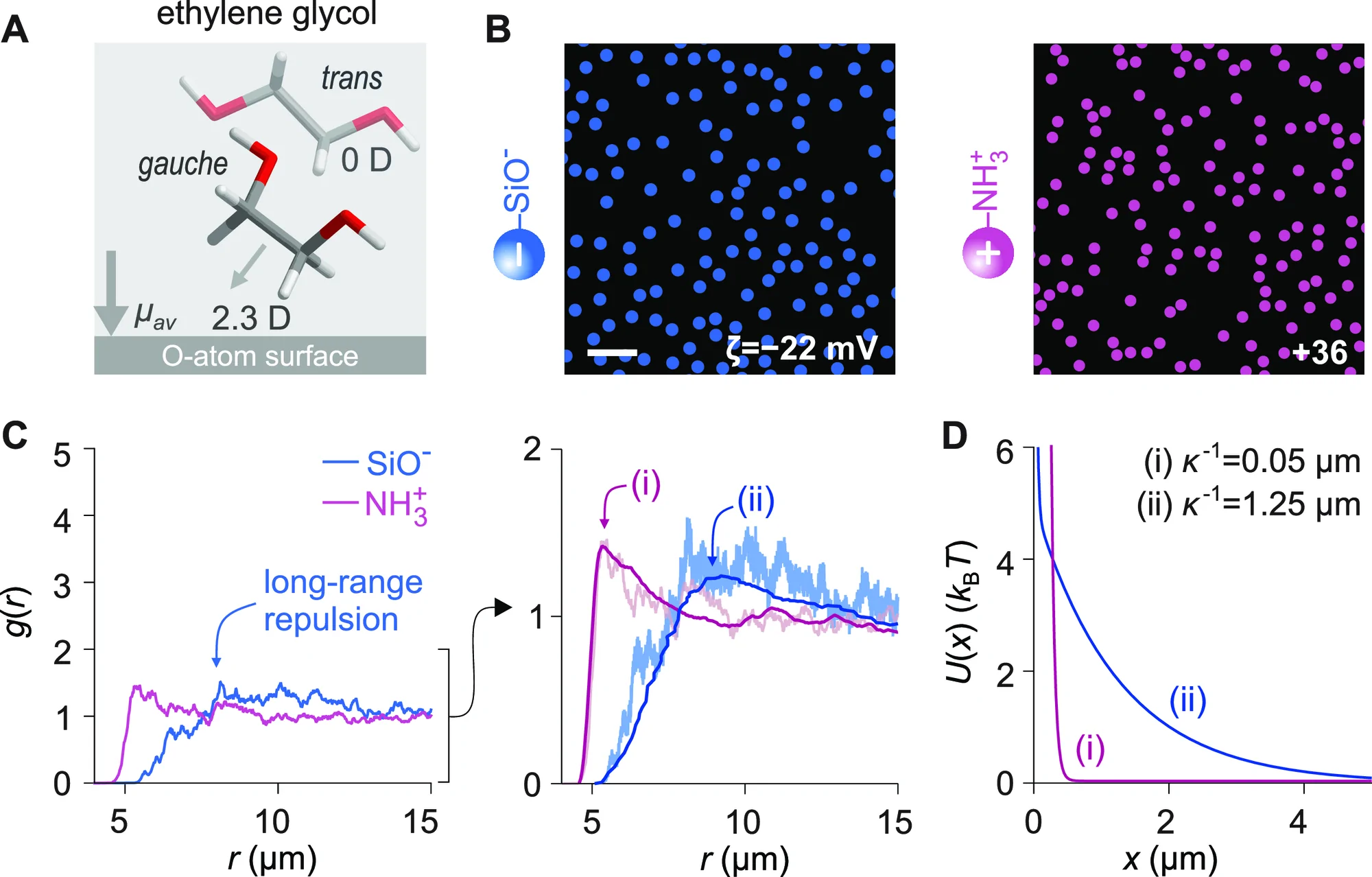
**Long-ranged repulsion in ethylene glycol. A** Molecular structures of trans and gauche conformers of ethylene glycol (EG), and schematic representation of the average molecular orientation at an uncharged oxygen-atom surface (molecular dipole moments – light gray arrows) as inferred from MD simulations. Normal dipole moment density, μ_av_, points toward the surface for the gauche isomer (dark arrow). **B** Digitised experimental images of two-dimensional colloidal suspensions of ca. 5 μm diameter negatively charged SiO_2_ particles (blue) and positively charged aminated silica (pink) in EG, neither particle type displaying the cluster-forming attraction over a wide range of p*H* from 1.2 to 9.5. Scale bar 20 μm. **C** Measured *g*(*r*) profiles indicate a long-ranged repulsion for negatively charged particles. For negative particles, the *g*(*r*) profile approaches ≈ 1 over a distance of a few micrometers (blue line), which is markedly different from the profile measured for positive particles (pink line). Right: Curves (i) and (ii) denote *g*(*r*) profiles computed based on trial interaction potentials *U*(*x*) described next (dark blue and pink lines). **D** Pair interaction potentials *U*(*x*) used as inputs to BD simulations that recapitulate the experimentally measured suspension structure: Curve (i) denotes *U*(*x*) = *A*exp­(- *κx*), where *A* = 10^3^ *k*_B_ *T* and κ^–1^ = 0.05 μm is the experimentally inferred Debye length. Curve (ii) denotes a trial potential of the form *U*(*x*) = *A*exp­(- *κx*) + 30exp­(- 50*x*), where A = 5 *k*_B_ *T*, and κ^–1^ = 1.25 μm is the inferred decay length of the interaction. The second sharply decaying, short-range repulsive term is included solely in order to prevent particle overlap in the simulation. Agreement between the simulated and measured *g*(*r*) profiles suggests that the interactions of positively charged particles in EG are well captured by the canonical electrostatic repulsion. However, a long-ranged repulsion characterized by a screening length κ^–1^ = 1.25 μm is required to capture the experimental behavior for negative silica particles in EG. Adapted from ref 8. Available under a CC-BY 4.0 license. Copyright 2025 Wang, S., Walker-Gibbons, R., Watkins, B., Lin, B., Krishnan, M.

Importantly, the experimentally inferred range of the repulsive interaction between 5 μm diameter negative particles in EG suggested a decay length of the repulsive force of ca. 1.25 μm (Figure 3D). This decay length is about 25 times longer than the canonical Debye screening length in the system, and is reminiscent of measurements characterizing the attractive electrosolvation force (Figure 8D). Positively charged particles in EG on the other hand display a repulsion with a decay length of ca. 50 nm which is consistent with the experimentally estimated Debye length and indicative of the canonically expected electrostatic interaction.

Moreover, in all experiments involving weakly acidic or weakly basic surface groups (e.g., -SiOH, −COOH, NH_2_), the p*H* was found to play a strong role in determining whether or not attraction occurred, regardless of whether the experiments were performed in water or in less polar media (Figure 4). In water for example, where the p*K* of the surface ionizable groups is well-known, attraction occurred in general when the solution p*H* lay in the vicinity of the p*K*_a_ of the ionizable groups. This feature may be contrasted with experiments involving particles coated with lipid bilayers, DNA and highly acidic polyelectrolytes^7,9^ (Figure 7A and B). Here the p*H* ≈ 5–6 of the solution lies significantly above the p*K*_a_ ≈ 1–2 of the ionizable groups, but strong interparticle attraction occurs nonetheless. Taken together the two families of observations might suggest that the balance of electrical charge, and a property reflecting the interfacial solvent structure, together determine whether the condition for long-range attraction is met. In the present thinking the interfacial solvent property driving long-range attraction or repulsion is ascribed to a “polarization charge”.^44,45^ Thus, in particle systems where electrical charge stems from weakly ionizable groups, p*H* seems to offer a knob that tunes the balance between electrical charge and interfacial polarization. However, there is a further important point to note here in that the structure and orientation of the interfacial solvent at a phospholipid bilayer and DNA-coated surfaces are expected to be significantly different from that at a charged silica or carboxylate particle surface.^69,78,79^ Yet all these systems display attraction and cluster formation behavior that is both qualitatively and quantitatively largely similar, as discussed in the following sections and in detail in ref 9. This observation points to the fact that crucial physicochemical features of the charge and solvent-dependent attractive interaction remain yet to be fully understood, as discussed later in the Theory section.

**Figure 4:**
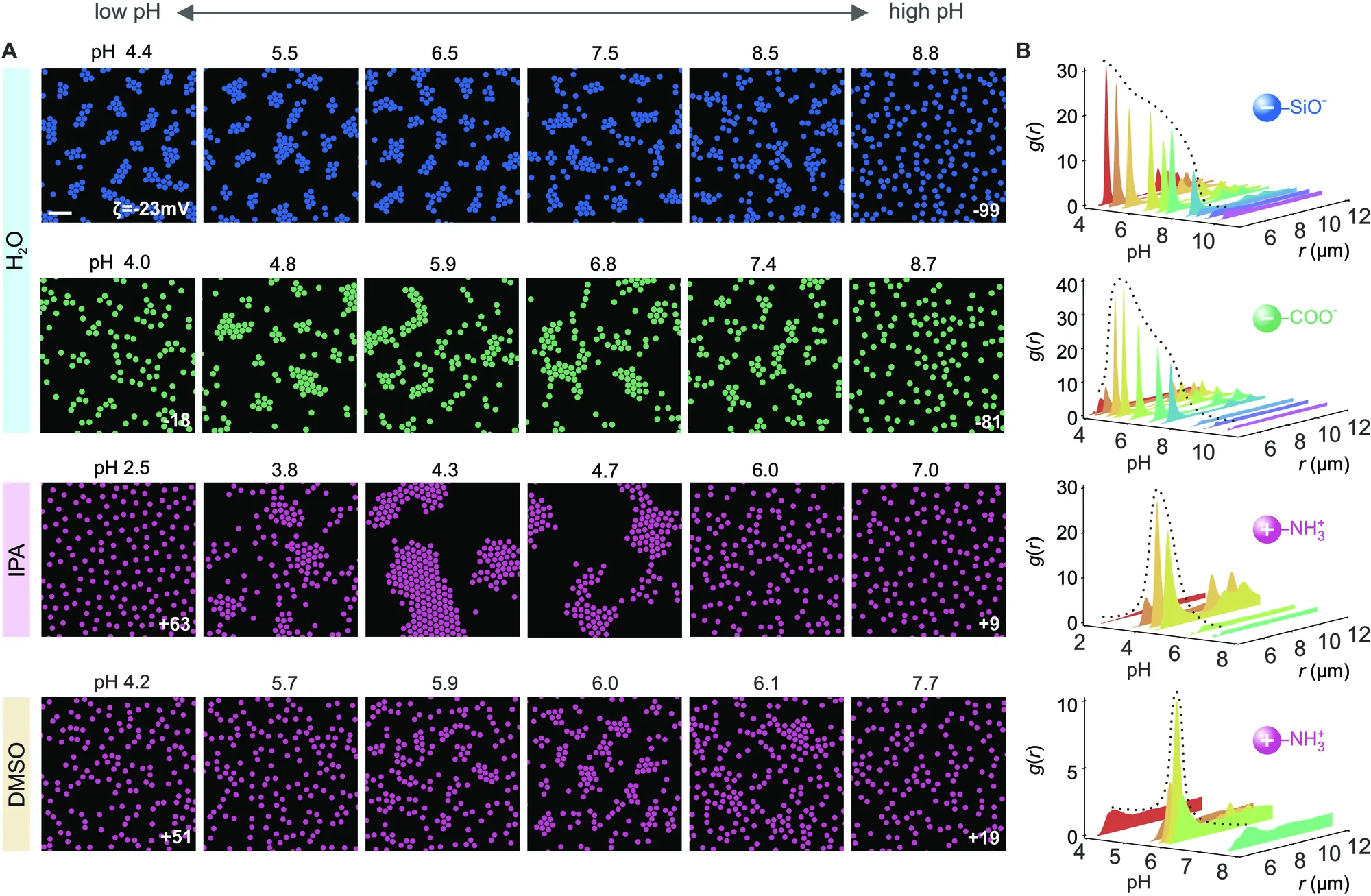
**The influence of p*H* on cluster formation in various solvents. A** Digitised microscopy images of ca. 5 μm diameter negatively charged SiO_2_ (blue) and COOH (green) particles in water, and positively charged aminated silica (pink) particles in IPA and DMSO, as a function of p*H* adjusted by the addition of HCl or Tris buffer to the medium (left). Measured zeta potentials of the particles are noted. Scale bar 20 μm. **B** Measured radial probability density function profiles, *g*(*r*), as a function of p*H*. Dashed envelope curves highlight the p*H* range where cluster formation is evident in each case. For negative particles in water, cluster formation occurs when the p*H* lies in the vicinity of the p*K*_a_ of the surface groups. Adapted from refs 7, 8 . Available under a CC-BY 4.0 license. Copyright 2024 Wang, S., Walker-Gibbons, R., Watkins, B., Flynn, M., Krishnan, M and Copyright 2025 Wang, S., Walker-Gibbons, R., Watkins, B., Lin, B., Krishnan, M., respectively.

## The Interparticle Attraction Can Be Modulated by the Properties of the Solid–Liquid Interface

### Solvent Mixtures

In order to specifically probe the dependence of the interparticle force on the properties of the interfacial electrolyte, we carried out two broadly different families of experiments.^8^ One set of experiments examined the interaction in solvent mixtures (Figure 5). Here we added water to suspensions of particles in anhydrous isopropyl alcohol and examined the effect on the interaction. We found that with increasing water content in the alcohol–water mixtures, the attraction between positively charged particles transitioned to a repulsion, and the repulsion between negative particles turned attractive (Figure 5A). We determined the concentration of water added to solvent required to abolish cluster formation for positive particles and to induce an attraction between negative particles. Since the surfaces in our experiments are hydrophilic, we expect water in the medium to adsorb preferentially to the particle surface as confirmed in MD simulations^8^ (Figure 5C). Thus, the properties of the interfacial solvent, e.g., the net normal dipole moment density, could be expected to be strongly influenced by the dominant solvent species at the interface. In particular, when the concentration of water added to alcohol exceeds a certain threshold, the interfacial solvent is expected to be dominated by water molecules. Indeed the attraction/repulsion signature observed in experiments followed expectations based on the sign of the normal dipole moment density of the solvent species expected to strongly populate the interface (Figure 5C). We further found that the percentage water content in alcohol required to turn a repulsion for negative particles into an attraction depended on the chemistry of the particle (e.g., silica or carboxylic acid particles), and even varied from one silica preparation to another (Figure 5B). These observations strongly implicate surface-chemistry dependent interactions with solvent molecules in the observed interparticle attraction.

**Figure 5:**
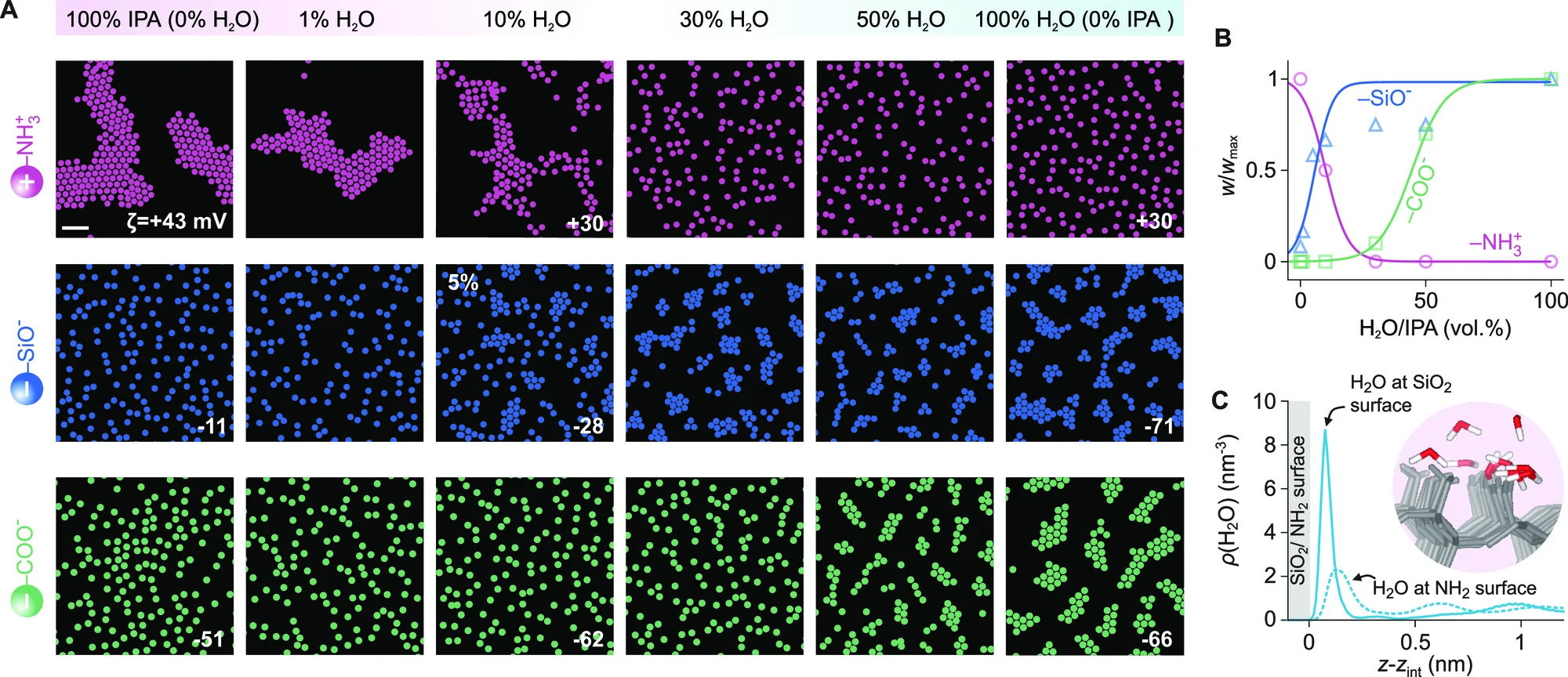
**Tuning the strength of the interparticle attraction in water-alcohol mixtures**. **A** Structure of two-dimensional suspensions of positively charged (pink), and negatively charged SiO_2_ (blue) and COOH particles (green), in water-IPA mixtures of increasing volume percentage of water (left to right). Positive particles display attraction and cluster formation in pure IPA containing water up to 10% (v/v), and repulsion at higher concentrations of added water (top row). SiO_2_ and COOH particle interactions are repulsive in pure IPA, but interparticle attraction and cluster formation set in at around 5% and 50% (v/v) water, respectively (middle and bottom rows). Measured zeta potentials, ζ, are noted. Scale bar 20 μm. **B** For each series of measurements, the normalized well depth, *w*/*w*_max_, in the inferred pair-interaction potential, is plotted as a function of water volume percentage to facilitate comparison. Lines are sigmoidal fits to the data. **C** Profiles of interfacial water density, ρ­(H_2_O), as a function of distance *z* from an interface situated at *z*_int_, as inferred from MD simulations of a 2% (v/v) water/IPA mixture in contact either with a silica (solid line) or a model amine surface (dashed line). Simulations indicate higher interfacial water densities relative to the bulk at both surface types. Inset: MD simulation snapshot of a 2% (v/v) water/IPA mixture, displaying the background medium IPA in pink and highlighting individual interfacial water molecules adsorbed to silanol groups on the silica surface. Adapted from ref 8. Available under a CC-BY 4.0 license. Copyright 2025 Wang, S., Walker-Gibbons, R., Watkins, B., Lin, B., Krishnan, M.

### Net-Neutral Molecules in the Electrolyte: Zwitterions, Osmolytes, and Surfactants

A related series of experiments probing the role of molecular species at the interface focused on adding small net-neutral solute molecules to the aqueous medium (Figure 6). These included polar and hydrophobic amino acids, osmolytes such as trimethylglycine (TMG) and trimethylamine N-oxide (TMAO), and surfactants such as Tween20 ^8^ (Figure 6). Many of these net-neutral species are expected to adsorb to the solid–water interface as has been verified independently in experiments on silica and mica, and in MD simulations.^80–85^ We set out to experimentally test the hypothesis that surface adsorbing species could be expected to perturb the interfacial solvent structure at a surface immersed in a pure solvent, and thereby modify the interparticle attraction. Amino acids are zwitterionic, net-neutral molecules and formally ought to have no impact on the interaction between two charged particles in solution, especially at low concentrations in the medium. But the experiments examining their impact on the cluster formation problem in fact showed the contrary: e.g., very low concentrations of tyrosine ≈ 100 μM abolished the electrosolvation attraction between silica particles entirely. Indeed we found that the concentration of amino acid required to mitigate the attraction decreased with increasing magnitude of adsorption energy of the amino acid to the glass–water interface. Thus, in order to abolish the attraction, a comparatively weakly surface-adsorbing amino acid such as Glycine was required to be present at approximately 1 mM in solution compared to sub mM concentrations for hydrophobic amino acids Tyr and Trp (Figure 6A and B). Further, surfactants such as Tween20 were found to exert a similar, attenuating effect on the long-range interparticle attraction. Tween 20 at concentrations of 1–2% in the medium (≈ 10 mM), typical for most practical applications, abolished cluster formation and turned the interaction between silica particles repulsive. This suggests that the widely known stabilizing effect of surfactants in staving off aggregation in matter suspended in solution may well rely on the ability of surfactants to alter (solvent) molecular-structuring at the solid–liquid interface and thereby weaken long-ranged solvation governed forces.

**Figure 6:**
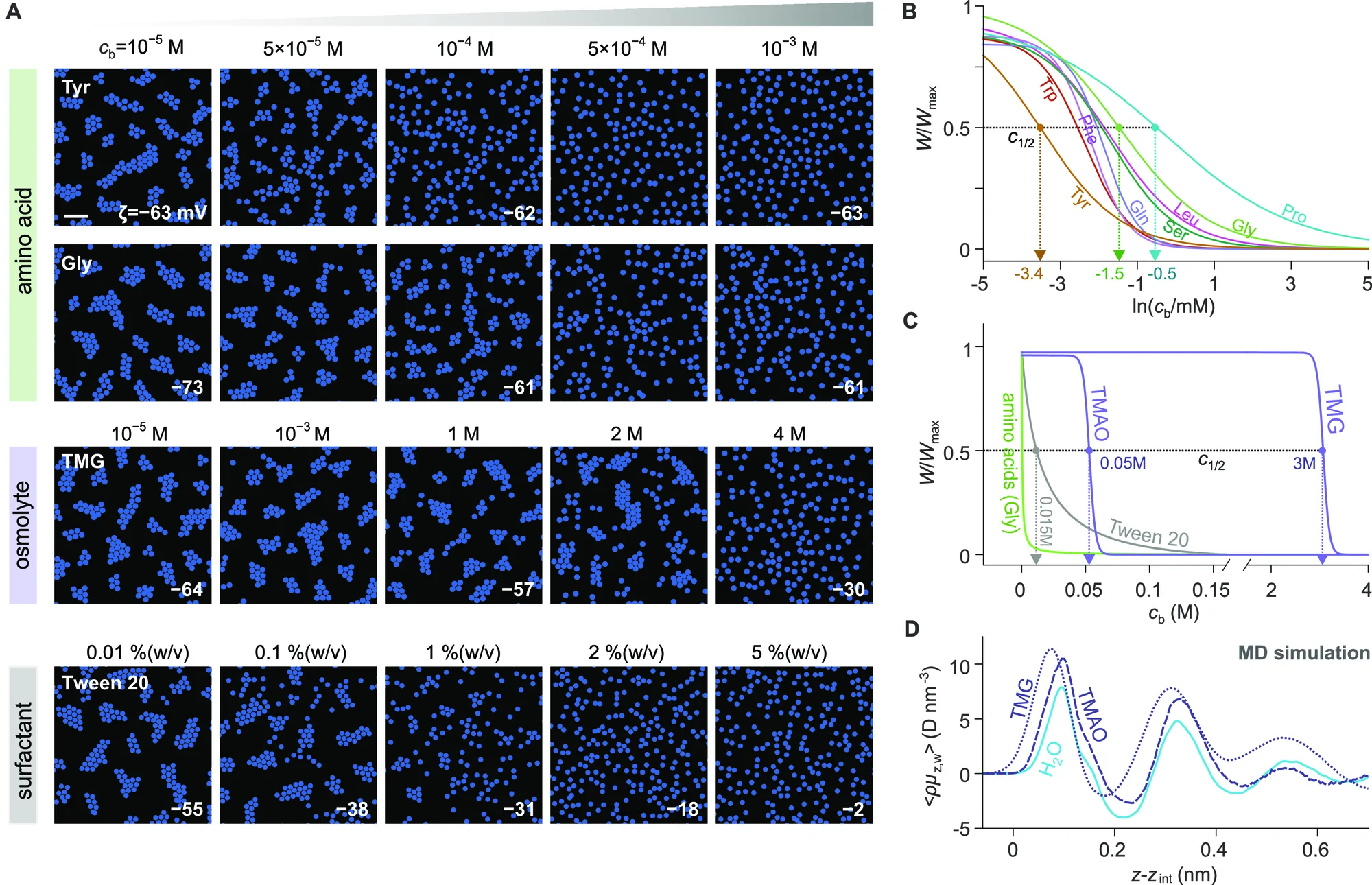
**The impact of small neutral molecules on the long-range interparticle attraction. A** Digitised images of negatively charged SiO_2_ particles in aqueous suspension containing various concentrations of net-neutral molecular species: zwitterionic amino acids and an osmolyte (trimethylglycine, TMG), or a surfactant (Tween20). In aqueous suspensions containing glycine and tyrosine, the strength of the interparticle attraction decreases significantly for amino acid concentrations of *c*_b_ > 200 μM for glycine and >30 μM for tyrosine in solution, and vanishes at higher concentrations. The attraction persists up to much higher concentrations for TMG (*c*_b_ ≈ 3 M), and 1% (w/v) or 8 mM for Tween 20. Scale bars 20 μm. **B** Normalized well depth, *w*/*w*_max_, as a function of *c*_b_ with sigmoidal fits to the data (colored lines) for all amino acids studied. The parameter *c*_1/2_ denotes the value of *c*_b_ at which the strength of the attraction is half-maximal, i.e., *w*/*w*_max_ = 0.5. **C** Normalized pair-interaction potential well depths, *w*/*w*_max_, for all categories of additives examined: zwitterionic amino acids, surfactants and osmolytes. **D** Spatial profiles of the normal component of the water dipole moment density at a silica interface for TMAO- and TMG-containing aqueous media (dashed blue lines) are consistently more positive than the pure water case (solid blue line), implying that the magnitude of μ_av_ of osmolyte-containing water is greater than μ_av_ for pure water. Adapted from ref 8. Available under a CC-BY 4.0 license. Copyright 2025 Wang, S., Walker-Gibbons, R., Watkins, B., Lin, B., Krishnan, M.

We also examined the effect of methylated zwitterions TMG and TMAO that are known to alter the structure and dynamics of water.^86–88^ In MD simulations, TMAO and TMG are observed to reinforce rather than disrupt the average orientation of interfacial water (Figure 6D). Qualitatively in line with these observations, we found that addition of trimethylated cationic zwitterions did not display any mitigating effect on the interparticle attraction up to molar concentrations in solution (Figure 6A and C). In fact particle clustering persisted at 3–4 orders of magnitude higher concentrations of trimethylated zwitterions than that at which amino acids abolished the long-range attraction.

The above experiments shared a common underlying theme in that they all perturbed the interfacial medium in a way that generally exerted a qualitative influence on the interparticle attraction. The effect of interfacially active molecules was generally disruptive to cluster formation, even at very low concentrations in the medium, often ≈ 10 ppm compared to the surrounding water. Intriguingly, trimethylated-cation carrying zwitterions that are known to reinforce structuring of interfacial water failed to disrupt cluster formation even at very high concentrations. It therefore seems rather likely that mechanisms related to solvent structuring at or close to the interface play an important role in the force driving cluster formation.

## Interparticle Attraction Mediated by Surface-Adsorbed Anionic Biological Polyelectrolytes

We also examined the interparticle interaction in mixtures of anionic particles possessing different surface chemistry. First of all, we made the striking observation that the property of long-range self-attraction was by no means restricted to particle chemistries traditionally available via chemical synthesis. Rather, anionic particles generated using layer-by-layer functionalization terminating with DNA and polyelectrolytes, as well as lipid bilayers, exhibited the same long-range, cluster forming behavior (Figure 7A-C). These observations mainly serve to highlight the generality of the interparticle attraction between anionic particles in water, emphasizing the rather secondary role played by specific surface chemistry in the overall physical effect.

**Figure 7:**
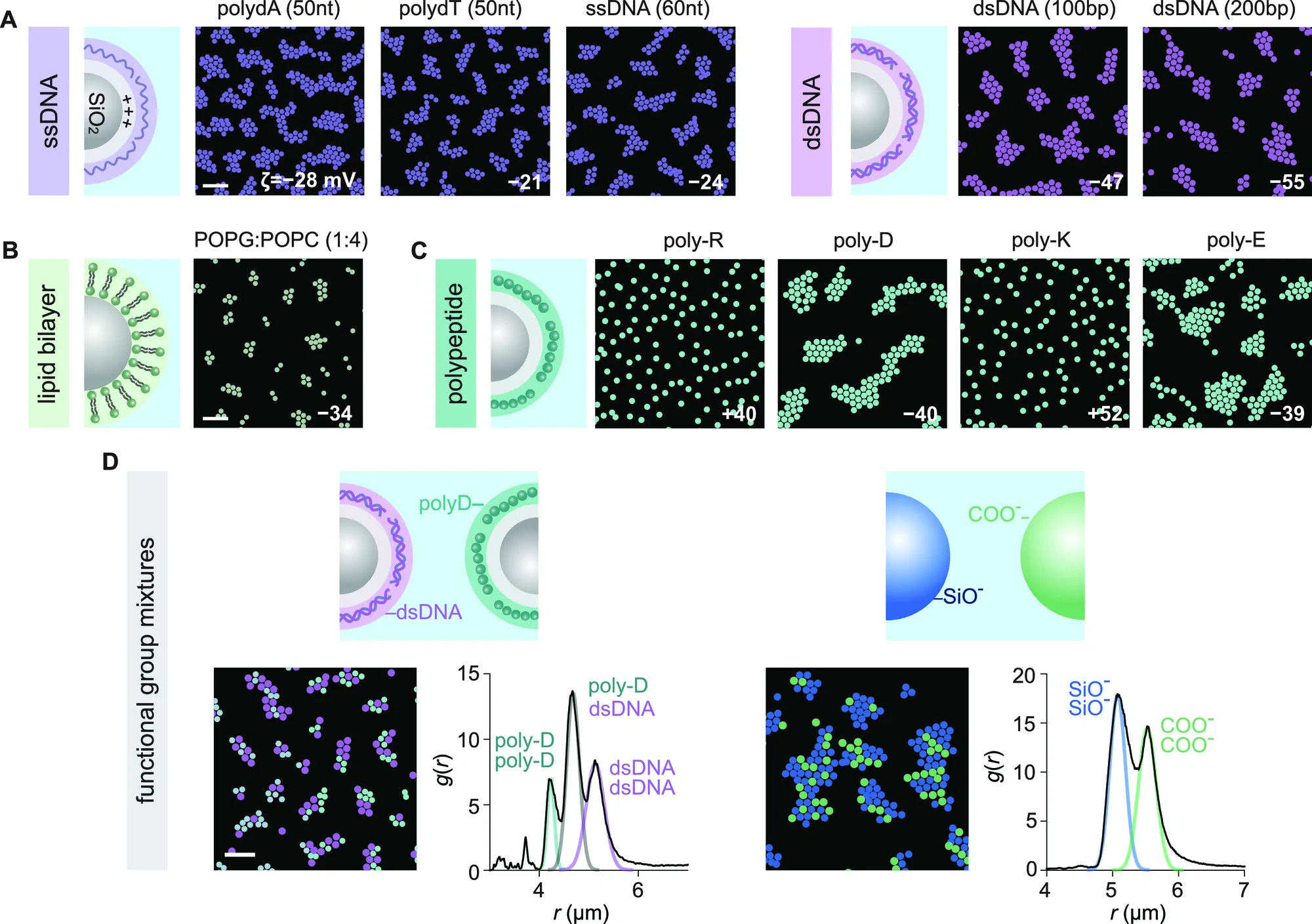
**Cluster formation in particles coated with anionic biomolecules: DNA, lipids and polypeptides.** Images depict cluster formation in suspensions of **A** SiO_2_ particles of diameter 3.9 μm functionalized with 50 nt poly dA, 50 nt poly dT, and 60 nt single-stranded (ss)-DNA using the positively charged polyelectrolyte (PDADMAC) as the first adsorbed layer in a two-step layer-by-layer deposition (left); and 4.8 μm diameter silica particles coated with 100 base pair (bp) and 200 bp double-stranded (ds)-DNA using the same procedure. **B** Cluster formation in aminated SiO_2_ particles 3.9 μm in diameter coated with net negatively charged lipid bilayers containing a 4:1 molar ratio of POPC to POPG (1-palmitoyl-2-oleoyl-*sn*-glycero-3-phosphocholine and -phosphoglycerol, respectively). **C** Interactions in SiO_2_ particles coated using layer-by-layer deposition of alternating layers of cationic (poly arginine (R) and poly lysine (K)) and anionic (poly aspartate (D) and poly glutamate (E)) polypeptides. Left: particles coated with alternating layers of poly-R and poly-D, where the coating of the outermost layer is noted. Right: poly-K and poly-E coated particles. While clustering is apparent for anionic particles, clusters do not form in suspensions of particles coated with cationic polypeptides. **D** Cluster formation in mixtures of anionic particles. Left: 100 bp dsDNA coated SiO_2_ (4.8 μm diameter) and poly-D coated SiO_2_ particles (3.9 μm diameter). The substructure of the first major peak in the measured *g*(*r*) profile reflects the three expected nearest neighbor separations. Right: Mixture of silica (4.8 μm diameter) and COOH particles (5.3 μm diameter), where the *g*(*r*) profile displays two of the three expected nearest neighbor distances. Scale bars 20 μm. Adapted from refs 7, 9 . Available under a CC-BY 4.0 license. Copyright 2024 Wang, S., Walker-Gibbons, R., Watkins, B., Flynn, M., Krishnan, M., and Copyright 2026 Wang, S., Le, A., Walker-Gibbons, R., Krishnan, M., respectively.

Furthermore, we have found that in order to exhibit attraction in water, anionic particles are not required to possess identical surface chemistry. Rather, particles with the same sign of charge but vastly different surface chemistry, e.g., silica and carboxylated microspheres, particles coated with DNA or anionic polyelectrolytes and polypeptides, all display “heterogeneous” cluster formation when mixed together: particles are incorporated into ordered clusters of heterogeneous composition regardless of their chemistry (Figure 7D). Thus, it appears that anionic matter in aqueous solution is poised to self-attract, and does so largely independently of the precise chemistry that confers negative charge.

The body of experimental work summarized above highlights a key attribute of the electrosolvation interaction, namely that the sign of the long-range force depends on both the sign and magnitude of the charge of the particle. This feature is accompanied by the corollary that the sign of the interparticle force for a given particle type can invert in a different solvent medium. Charge-asymmetric and solvent dependent cluster-formation seems to recapitulate features known from charge-asymmetric ion solvation, which suggests a direct link between interactions in solution and the properties of the interfacial solvent.

## Spatial Profile of the Pair-Interaction Potential: Measuring the Range of the Attraction

A final experimental feature that merits careful consideration concerns the spatial range of the attractive interaction that drives particle clustering. To address the range and spatial properties of the attractive force we have performed a series of direct measurements of the interaction potential on isolated pairs of particles using optical microscopy^9^ (Figure 8). In these measurements, we observed pairs of attracting particles engaged in “bound states” over durations of tens of minutes and constructed radial probability density functions *P*(*r*) of the interparticle separation *r* (Figure 8B, C). “Boltzmann inversion” of a measured probability density function yielded the spatial profile of the underlying pair potential *U*(*x*) as a function of intersurface separation *x* (Figure 8C, right panel). Examining an isolated pair of particles rules out “many body” contributions from neighboring particles and fosters the measurement of a true pair interaction potential.

**Figure 8:**
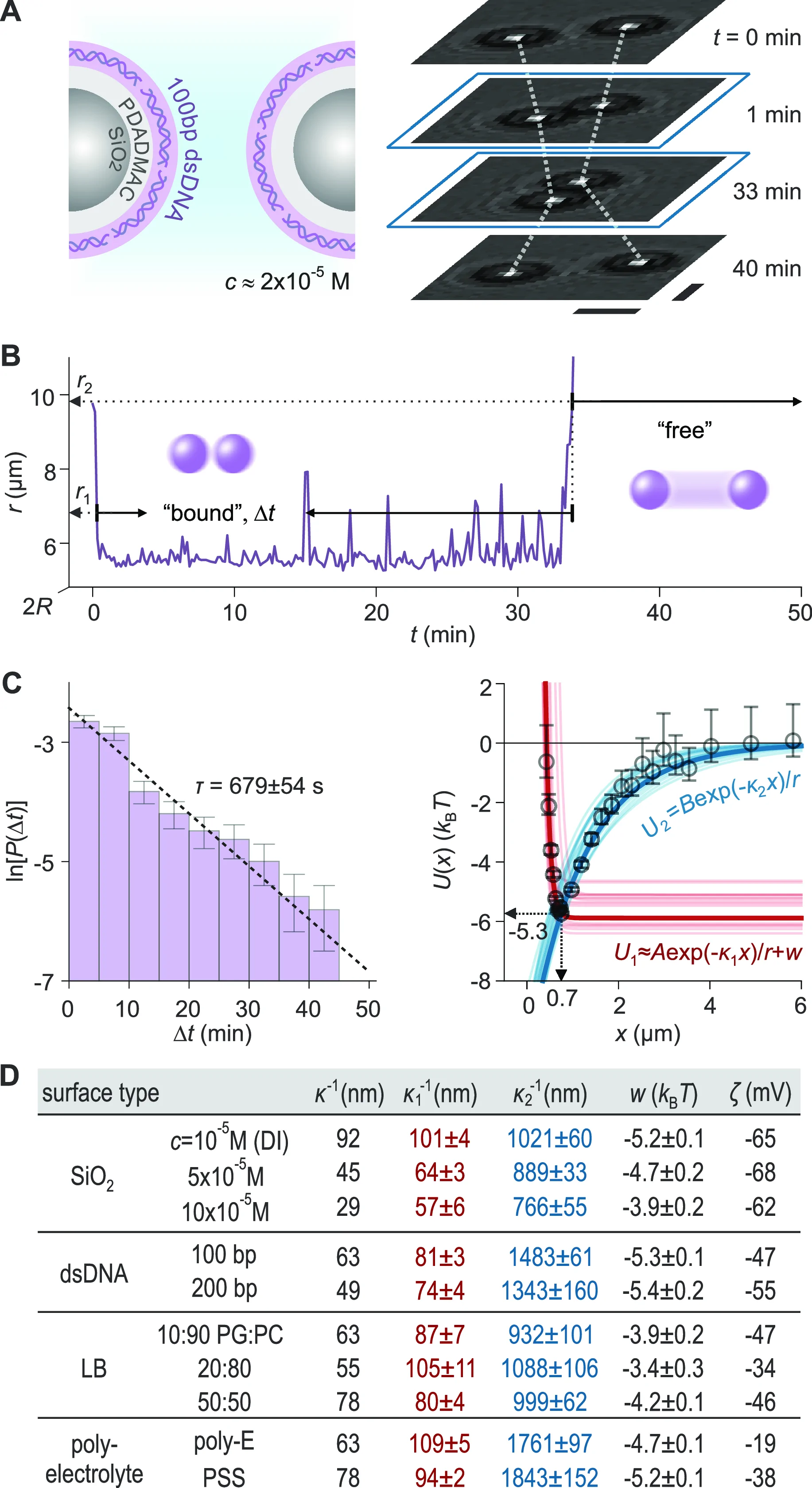
**Direct measurements of the pair interaction potential for two attracting DNA-coated particles**. **A** Schematic representation of silica particles (4.8 μm diameter) coated with 100 bp dsDNA interacting in water (left). Time-series of images for a pair of particles displaying “bound” (blue frames) and “free” states (right). Scale bars: 5 μm. **B** Time-dependent interparticle separation *r* for particles engaged in a bound-state. The start and end of a bound state are characterized by *r* < *r*_1_ and *r* >*r*_2_, which occur at *t* ≈ 1 min and *t* ≈ 33 min in the displayed trace, respectively. **C** Histogram of bound-state durations for 282 pairs of particles with fits of the data to the form *P*(Δ*t*) = (1/τ)­exp­(Δ*t*/τ), where Δ*t* is a measured bound-state duration for a single pair and τ is the average lifetime (left). Exponentially decaying piecewise fits of the attractive and repulsive arms of the potential for 21 pairs of particles (thin blue and red lines, respectively). Also presented are data for a representative particle-pair (symbols) and corresponding fits to the measured data (thick lines) with fitted decay lengths noted in **D**. **D** Table listing the nominal Debye length, κ^–1^, fitted parameters κ_1_^–1^, κ_2_^–1^ and *w*, and ζ-potentials for several anionic surface coatings: dsDNA, lipid-bilayers (LBs), polyglutamic acid - poly-E, and polystyrenesulfonate – PSS, which can be compared with measurements for uncoated silica particles at three ionic strengths. Pair potential parameters are averages over measurements on 9–21 pairs of particles in each case. Errors denote s.e.m (standard error of the mean) on fitted values across all particle pairs in a given data set. Adapted from ref 9. Available under a CC-BY 4.0 license. Copyright 2026 Wang, S., Le, A., Walker-Gibbons, R., Krishnan, M.

Direct measurements on pairs of particles in the diameter range 3–5 μm, possessing a variety of surface chemistries such as silanol groups, polyelectrolytes, DNA and anionic phospholipid LBs, have furnished important insight into the spatial properties of the pair interaction. While we have found that the length scale determining the shorter-range repulsive arm of the interaction κ_1_^–1^ is well approximated by the Debye length κ^–1^ in eq 1, the range of the attractive domain of the interaction has been found to be about an order of magnitude larger, with κ_2_^–1^ ≈ 1–2 μm (Figure 8C, D). The only obvious length scale in the system of a similar order is the particle radius itself. It is worth mentioning in this context that early reports on crystallite formation in sedimented negatively charged Tipula Iridiscent Virus in water noted a similar characteristic: the measured intersurface separation between virus particles in clusters was about 50–70 nm, which is comparable with the radius of the hydrated virus particle of approximately 90 nm.^12^ This observation may be compared with minima occurring at about 1 μm separation for particles of ca. 2.5 μm radius reported in our measurements.^7,9^ Note that DNA and polyelectrolyte coated particles exhibit even longer ranged attraction compared to silica and LB-coated particles, possibly hinting at a role for surface structural wavelengths in the interaction^89^ (Figure 8D). Taken together the observations prompt consideration of whether the size of the “defect” embedded in the medium – i.e., the particle in the electrolyte – can imprint a corresponding length scale on the interaction. Although necessarily speculative, these hypotheses may play a valuable role in shaping future efforts to formulate a comprehensive mechanistic model of the long-ranged attraction.

## Practical Applications of the Electrosolvation Force

To conclude the section on experimental investigations, we briefly touch upon possible technological applications of the electrosolvation interaction. Regardless of how well the underlying physics is currently understood, the ability to manipulate interactions between molecular level building blocks in solution raises prospects for applications across scales, in areas as diverse as colloidal self-assembly, molecular-level optical genome mapping and antifouling/smart surface coatings.

Some preliminary examples of progress in this direction are shown in Figure 9. Guided by the simple existing models of the electrosolvation force we have begun to establish empirical rules that could direct systematic practical exploitation of the interparticle interaction. The pH in solution, particle size and surface chemistry can be used to create heterogeneous clusters with different morphologies, early examples of which are shown in Figure 7. Clearly optical manipulation of individual particles may be used in conjunction with the electrosolvation interaction to drive spatially controlled assembly of micron scale matter in suspension.

**Figure 9:**
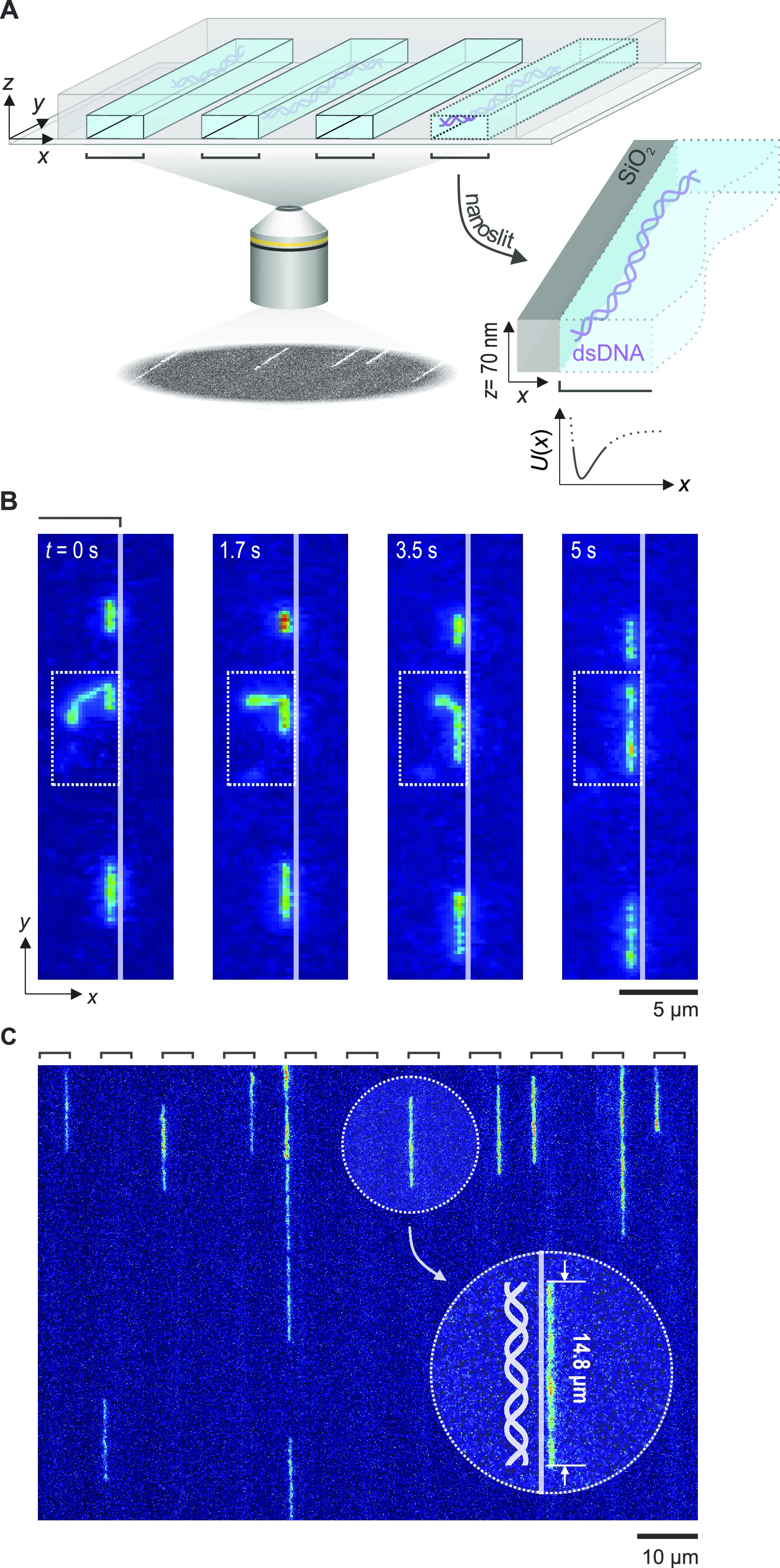
**A practical application of the electrosolvation attraction. A** Schematic representation of fluorescence microscopy experiments used to observe the attractive interaction between single fluorescently labeled λ-DNA molecules and the effectively one-dimensional sidewall of a two-dimensional glass-silicon dioxide nanoslit (not to scale).^33^ Slits are 5 μm wide and 70 nm deep. Black braces denote locations of the nanoslits. **B** Time-series of images displaying short fragments of λ-DNA (enclosed in box) progressively drawn into a putative one-dimensional potential well along the sidewall of a 100 nm deep nanoslit (vertical white line). Data reproduced with permission from ref 33. **C** Attraction between negatively charged DNA and like-charged silicon dioxide sidewalls of glass/silica nanoslits of depth 70 nm can be strong enough to stretch DNA. λ-DNA molecules are nearly fully stretched at high density to an average length of 14.5 ± 0.1 μm which is about 90% of the nominal contour length of 16.5 μm, as highlighted in the inset. In this experiment λ-DNA was labeled using the intercalating cyanine dye POPO-3 at a dye:basepair ratio of 1:10, which increases the overall contour length of the DNA. The experiment was performed in deionized water containing 0.1 mM Tris. Stretched molecules can be translocated by flow through the field of view. Adapted from ref 33. Copyright 2007 American Chemical Society.

Next, previous reports on spontaneous stretching of DNA in nanofluidic slits very likely represented an early technologically relevant manifestation of the electrosolvation attraction (Figure 9). Here, fluorescently labeled DNA molecules (λ-DNA) introduced into silicon dioxide-glass nanoslits, 100 nm or less in depth, stretched out in one-dimension along the sidewalls of the slit.^33,35^ The loss in configurational entropy was evidently compensated by an energetically favorable interaction between the DNA and the silica wall. Although the physics behind the putative attraction was poorly understood at the time of the observations, it is possible that the origin of the attraction between negatively charged DNA and the like-charged silicon dioxide sidewalls of the nanoslit lies in the electrosolvation force. The hydration structure surrounding the DNA double helix is highly complex. It reflects contributions from both the strongly anionic phosphate groups along the backbone and the structured water networks within the groove regions, including the well-known hydration spine of the minor groove.^78,79^ As a result, solvent orientation around DNA is expected to be highly heterogeneous and strongly site-dependent. Assuming for simplicity that the dominant contribution to the polarization around DNA originates from the phosphate-rich backbone, then interactions between strongly charged DNA and less strongly charged, and likely heterogeneous, silica sidewalls could reflect a scenario illustrated in Figure 11 (panel (vi)), which would give rise to an attractive electrosolvation force. However, given the complexity and heterogeneity of DNA hydration, we must regard such a mechanistic interpretation as tentative and speculative at present. Regardless, the attraction driving spontaneous DNA stretching could find use in optical genome mapping experiments that rely on DNA molecules stretched at high density for fluorescence-based imaging of modifications along the polymer contour.^90^ The chip-based nanoslit format of the experimental setup used to achieve spontaneous DNA stretching in solution lends itself well to high-throughput optical genome mapping studies.

Finally, it is worth considering whether attractive electrosolvation forces may contribute to colloidal flocculation in situations where the formation of clusters, flocs and aggregates are conventionally attributed primarily to van der Waals interactions. While the relative importance of these mechanisms remains to be established, the possibility that interfacial solvent polarization contributes to colloidal stability and aggregation warrants further investigation. Given the central role of flocculation in a wide range of industrial and environmental processes, a deeper understanding of the underlying interaction mechanisms could offer new opportunities for controlling colloidal stability. In a similar vein, while the examples reviewed here focus largely on tailoring and harnessing the electrosolvation attraction, the repulsive form of the interaction also serves important practical applications. E.g., the powerful yet largely poorly understood role played by zwitterionic coatings in preventing biofouling could prove to be a significant instance of how the repulsive electrosolvation force may find further practical relevance.^91,92^

The following section summarizes the current state of understanding of the theoretical underpinnings of the observed solvent governed interparticle force. The section thereafter explores possible alternate explanations for the experimental observations. These discussions are included in this review for completeness on the current state of thinking on the topic, and do not constitute essential reading.

## Theoretical Descriptions of the Solvation Governed Interparticle Interaction

### A Preliminary Model of Particle Attraction Due to Interfacial Solvation

Beyond a thorough exploration of the electrosolvation interaction at the experimental level we have also sought to contribute to a formal understanding of the physical mechanism(s) underlying the observations using both MD simulations and theory. Our initial theoretical model of the interaction provided a preliminary “effective” description of the force, and successfully captured a surprisingly large number of features of the interaction.^4^ But this model of the interparticle free energy had a hybrid character in that it separately treated the electrostatics within a mean field description, and then added in an estimate of the interfacial solvation free-energy contribution, inferred from MD simulations, to arrive at the total free energy.^4^ The superposition of the two contributions resulted in an early indication that the total interaction energy could be given by the sum of two exponential terms such that^5^

1 $$U_{\mathrm{tot}}=\Delta F_{\mathrm{el}}+\Delta F_{\mathrm{int}}\approx A\exp\left(-\kappa_{1}x\right)+B\exp\left(-\kappa_{2}x\right)$$

Here, the first term, Δ*F*_el_ = *A*exp (-κ_1_ *x*) > 0, represents the familiar electrostatic repulsive interaction free energy between two large like-charged spheres of radius *R* in the regime *R* ≫ κ^‑1^ = κ_1_^‑1^. Note that *A* is always positive, capturing the traditionally expected repulsion between electrically like-charged objects. The second term Δ*F*_int_ ≈ *B*exp (-κ_2_ *x*) represents the interfacially generated contribution to the interaction of two objects in solution as suggested by the interfacial solvation model.^4,5^ Within this model we found that κ_2_ < κ_1_, and

2 $$B\propto z\mu_{\mathrm{av}}\left(\sigma \right)$$

where *z* is the sign of the charge carried by the particle and μ_av_ (σ) was the excess normal (solvent) dipole moment surface density at the interface between an object of surface charge density σ and the electrolyte, which can be obtained from MD simulations.^8^ Note that unlike *A*, eq 2 shows that the sign of *B* can be positive or negative. Thus, depending on the sign and magnitude of *B* in the “electrosolvation term”, the interaction between electrically like charged particles in solution could be expected to be either net-attractive or repulsive at long-range. Importantly the model indicated that this additional interfacial term could produce a long-ranged minimum in the pair-potential provided *B* is sufficiently large and negative in sign. We note on the side that as discussed in ref 8, μ_av_ ∝ - φ_int_ where φ_int_ or φ_0_ is the excess electrical potential due to oriented solvent molecules at an interface.

The interfacial solvation model represented a preliminary attempt to explain the experiments and was able to capture several key features of experiment, including (1) the asymmetry of the sign of the long-range force with respect to particle charge in a given medium, (2) the inversion in nonaqueous media of the charge-asymmetric trend observed in aqueous solutions, as well as (3) the p*H* dependence and salt concentration dependence of the attraction. Despite these key successes, this highly simplistic model harbored a few important limitations. One of the immediately evident shortcomings of the model was its inability to recover the shorter-ranged repulsion observed in experiments performed at variable p*H*.^7^ A strong repulsion on close approach (interparticle separations of <5κ^–1^) is not only characteristic of the canonical interaction between like-charged particles, but is in fact also observed in all experiments where particle clusters form.^7,9^ But, except at the lowest salt concentrations, our preliminary model generally predicted attraction at shorter separation, suggesting monotonic approach of the particles to contact which was not seen in the experiments. Furthermore, self-attraction observed in later experiments on a variety of highly acidic systems of lipid-bilayer, polyelectrolyte and DNA-coated particles seemed unlikely to fully align with the framework of the original model. One reason is that the model required the approaching particles to charge-regulate in order to trigger the interfacial solvent contribution to the free energy. But at p*H* values 5–6 units above their p*K*_a_ values, highly acidic surfaces are unlikely to charge-regulate to an extent that is sufficient to trigger a substantial separation-dependent free energy contribution from the interfacial solvent. This calls into question the crucial importance of charge regulation in the originally proposed mechanism. A second, more subtle, yet arguably vital point concerns the precise role and contribution of the interfacial solvent. The solvation structure at DNA and phospholipid-bilayer interfaces is spatially nonuniform and strongly influenced by the high density of highly acidic phosphate groups. Water molecules around these highly anionic groups orient with their oxygen atoms pointing toward the bulk: opposite to their net orientation at weakly charged or indeed near neutral surfaces for which the model was originally constructed.

A final, more obvious limitation of the original model concerned the magnitude of the inverse screening length κ_2_ in eq 1. The model suggested a value for κ_2_^–1^ a few percent larger than κ_1_^–1^ ≈ κ^‑1^, the Debye length.^5^ This does not align with the extremely long decay length that has since been directly measured for the attractive force^9^ (Figure 8). However, the model was inherently simple, and relied on an additive correction to the standard Poisson-Boltzmann theory arising from anisotropic behavior of the interfacial solvent. This framework held no room for any other length scales, e.g., those that stem from possible correlations in the medium or surface structural wavelengths, as discussed further below.^89^

### A Nonlocal Electrostatics Model of Interactions in the Fluid Phase: Background

In search of an improved theoretical model of the interaction we have undertaken a further step, aiming to satisfy two major requirements: to (1) eliminate the “patchwork” character of the original model in favor of a more self-consistent framework that directly incorporates the behavior of the interfacial solvent, and (2) integrate into the model a microscopic description of the bulk solvent medium.

Since the 1970s the theoretical literature has discussed the implications of the discrete, molecular nature of water in the solvation of ions.^93^ The broad framework within which such behavior is discussed goes under the name of “nonlocal” electrostatics. These models incorporate the ingredients necessary to capture departures in the properties of correlated media such as real solvents from that expected for an idealized continuous medium characterized by a uniform dielectric constant. Thus, in a ‘local medium’, the solvent is regarded as a ‘structureless, shielding background’ where the local polarization depends only on the electric field at the same point. In contrast in a ‘nonlocal’ medium (likely to describe most solvent media), the polarization at a point can depend on the electric field at any other distant point. Correlations in the medium, arising, e.g., from nearest neighbor dipole–dipole interactions, or hydrogen-bonding interactions in the case of water, can give rise to nonzero length scales characterizing the spatial rate of decay of a perturbation to the local polarization. Theoretical work from the 1980s and earlier, has suggested that these correlations could give rise to markedly different, “non-DLVO” behavior from that expected of the canonical, correlation-free medium.^94^ Thus, the nonlocal electrostatics view suggests that the decay length scale of the electrical field and electrical potential could carry a key contribution from a polarization correlation length scale, ξ.

Previous work has also demonstrated that the nonlocal view can shed light on the origin and characteristics of the short-range hydration force. Thus, nonlocal models accounting for nearest neighbor dipole–dipole interactions in water were sufficient to capture the ∼ 1.5 Å decay length of the short-range hydration force which emerged from the assumptions of the model.^95^ However, earlier more generalized models that accounted, e.g., for Bjerrum defects in the medium, treated ξ as a tunable parameter.^96^ In fact real systems, can be far more complex, involving contributions from surface structural wavelengths, and it is not clear what the parameter ξ should be, and indeed if it is even correct to speak of a single length scale.^89^ Nonlinear spectroscopy measurements over the last couple of decades have suggested very long ranged decay of orientational polarization in water, with measured length scales ranging from 5 to 25 nm, or even up to 100 nm in liposomes – orders of magnitude larger than the 3–4 Å molecular length scale.^97–99^ Such insight ought to have profound consequences for interactions in the solution phase along the lines of the evidence emerging from the experiments reviewed here.

### Key Insights into the Electrosolvation Interaction from a Nonlocal Electrostatics Perspective

Our preliminary effort to construct a nonlocal model of electrostatic interactions now offers opportunities for fresh insight into interactions in the fluid phase.^44^ In particular, models of nonlocal electrostatics naturally yield multiple screening lengths which is a significant departure from the rather fixed notion of the screening (Debye) length in DLVO theory.^44,94–96,100^ These models suggest that the effective screening length κ_eff_^–1^ relevant for long-range interactions is not necessarily identical to the Debye length but can in fact be significantly augmented from that value by the correlation length of the solvent medium ξ. Thus, the longest screening length in an electrostatic interaction can be given by

3 $$\kappa_{\mathrm{eff}}^{-1}\approx \sqrt{\kappa^{-2}+\frac{\varepsilon_{s}}{\varepsilon_{\infty }}\xi^{2}}$$

where *ε*_s_ and *ε*_∞_ are the values of the dielectric function at low and high spatial frequencies, taking typical values of 80 and 2-5 respectively.^44^ For a Debye length κ^–1^ = 1 nm and ξ = 1 nm, the nonlocal view envisages an effective screening length of the order κ_eff_^–1^ ≈ 4–7 nm, much longer than the nominal Debye length. In fact, nonlocal models naturally deliver the possibility of solvent-mediated attraction between electrically like-charged objects such as proteins, or even DNA, under physiological conditions as briefly discussed later. Importantly, we have further found that in a nonlocal medium, both interfacial polarization, as well as structural dipoles at an interface, can produce spatially decaying electrical potentials that propagate into the far-field.^44^ Such electrical signatures would be indistinguishable from those created by electrical charge (Figure 10). The electrical potential due to an object in solution may now contain significant contributions from both its electrical charge and interfacial polarization, even at significant distances, which carries important implications for interparticle interactions (Figure 10).

**Figure 10:**
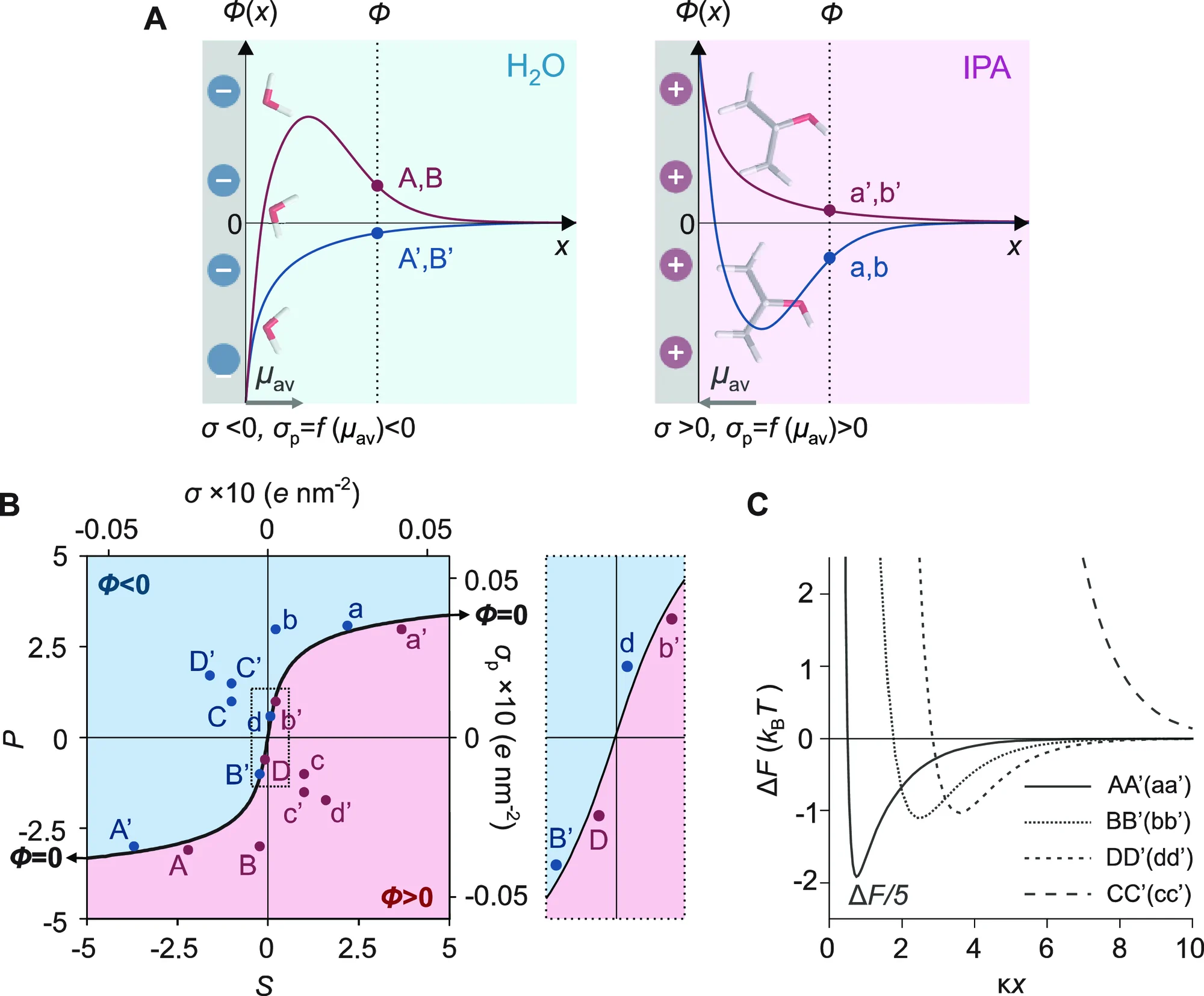
**A nonlocal electrostatics model of interparticle interactions captures experimental features**. **A** Illustration of a key concept in our nonlocal electrostatics model: both electrical charge on a surface as well as interfacial polarization due to structured solvent molecules at the interface may contribute to the spatial electrical potential profile ϕ­(*x*) in the medium. The contribution from the polarization may either reinforce or counteract that due to the charge. Depending on the signs and relative magnitudes of the fixed charge density σ and the polarization charge density σ_p_, the surface may generate a net potential ϕ of positive or negative sign at large distances *κx* ≥ 3 (dashed vertical line). Left: Sketches of potential profiles for surfaces with σ < 0 and σ_p_ < 0, as given by the points *A* or *B* (red) and *A’* or *B’* (blue) in the plot in panel B. Schematic representation of interfacial water molecules depicts the orientation expected at a weakly charged or neutral surface. Right: Qualitative potential profiles for surfaces with σ > 0 and σ_p_ > 0, as given by the points *a* or *b* (blue) and *a’* or *b’* (red) in panel B. Molecular interfacial orientation depicts isopropanol (IPA) at a neutral surface. **B** Two-dimensional plot of the sign of ϕ at a large distance from a surface whose electrical charge and polarization are given by σ and σ_p_. The black contour denotes ϕ = 0 and separates regions of ϕ > 0 (red) and ϕ < 0 (blue). The calculation corresponds to κ^–1^ = 100 nm, ξ = 10 nm, *ε*_s_ = 80 and *ε*_∞_ = 5. **C** Interaction free energy profiles for pairs of 5 μm diameter spheres whose charge and polarization densities are given by the points marked in **B**. A pair of interacting surfaces with properties on opposite sides of the zero-potential contour (black curve) generally yields a minimum in the pair potential. Pairs of the surfaces on the same side of the contour always yield monotonically repulsive behavior. The model can therefore recapitulate monotonic repulsions that are qualitatively in line with Poisson-Boltzmann theory (e.g., curve CC’), as frequently noted in experiments, or indeed long-range attractive potentials with minima occurring at variable intersurface separations (e.g., curves AA’, BB’, DD’). Because in water, MD simulations suggest σ_p_ < 0 for weakly charged surfaces, we may expect attractive electrosolvation forces and a potential minimum for negatively charged surfaces (e.g., curves AA’ and BB’), and repulsion for positive surfaces (curve cc’). The converse holds for particles in alcohols where we expect σ_p_ > 0, and therefore attraction for positive particles (e.g., curves aa’, bb’) and repulsion for negative particles (e.g., curve CC’).^45^ Data for curve AA’ has been divided by 5.

Thus, in summary, there are three significant points of departure between the nonlocal electrostatics theoretical framework described in refs 44, 45 and our previous simpler model of the electrosolvation interaction in ref 4. The nonlocal framework (1) clearly supports electrical signatures in the far field arising from surface polarization due to the solvent, where PB theory in a local medium does not, (2) explicitly invokes additional length scales – the polarization correlation length, ξ – which offers the possibility of effective screening lengths that are longer than the Debye length, and (3) does not admit attraction between identical and homogeneous particles. The nonlocal description contains an additional differential equation for the polarization, which in turn permits an additional boundary condition specifying the value of the polarization charge density σ_p_ at the interface (Figure 10B). This possibility is not offered in a local medium where the polarization is fully defined by the local electric field, and vanishes for zero electric field. In our nonlocal model, for an uncharged interface, the relationship between σ_p_ and the excess interfacial dipole moment density per unit area, μ_av_, from MD simulations may be given by σ_p_ ∼ - μ_av_/*l*, where *l* = O­(ξ) is the thickness of the interfacial region.

Importantly, our current nonlocal electrostatics model only supports attraction between particles that are either nonidentical, or, identical yet heterogeneous. Thus, within the present theoretical view, interparticle attraction requires like-charged objects to carry surface heterogeneity in either their charge or interfacial polarization or both. Such heterogeneity ought to be typical for naturally occurring molecular and particulate matter.

### “Antisymmetric” Interactions Can Drive Attraction between Similarly Charged Particles

Faces of approaching objects characterized by nonidentical properties may engage in what we may term an “antisymmetric” interaction (Figure 10B, 11).^44,45^ Here the sign and magnitude of the particles’ net electrical charge for example could be similar or even identical, but dissimilar values of surface polarization would be sufficient to trigger an attraction. The properties of pairs of particles that attract at long-range, despite carrying similar electrical charge, are given by points lying on opposite sides of the zero-potential contour in Figure 10B. Some examples are noted using the format (B, B’) or (b, b’). Analogously, particles with similar interfacial polarization values but unequal charge of the same sign could also yield the same effect, e.g., (A,A’) and (a,a’). Here, a comparatively larger contribution of the interfacial polarization to the “far-field” electrical potential for one isolated particle compared to its like-charged interaction partner results in potentials of opposite sign to each other. This sets up the “antisymmetric” character of the interaction between two particle faces, meeting the condition for a long-range attraction. Unlike fixed surfaces approaching each other – e.g., in mechanical force measurement experiments – particles and molecules in solution possess orientational degrees of freedom that can be sampled on timescales comparable to translational motion. Therefore, two like-charged particles carrying surface heterogeneity in their charge and/or interfacial polarization, and approaching each other from infinite separation, could naturally reorient to engage in an interaction that minimizes the interaction free energy of the system, permitting long-range attraction depending on the particle properties.

**Figure 11:**
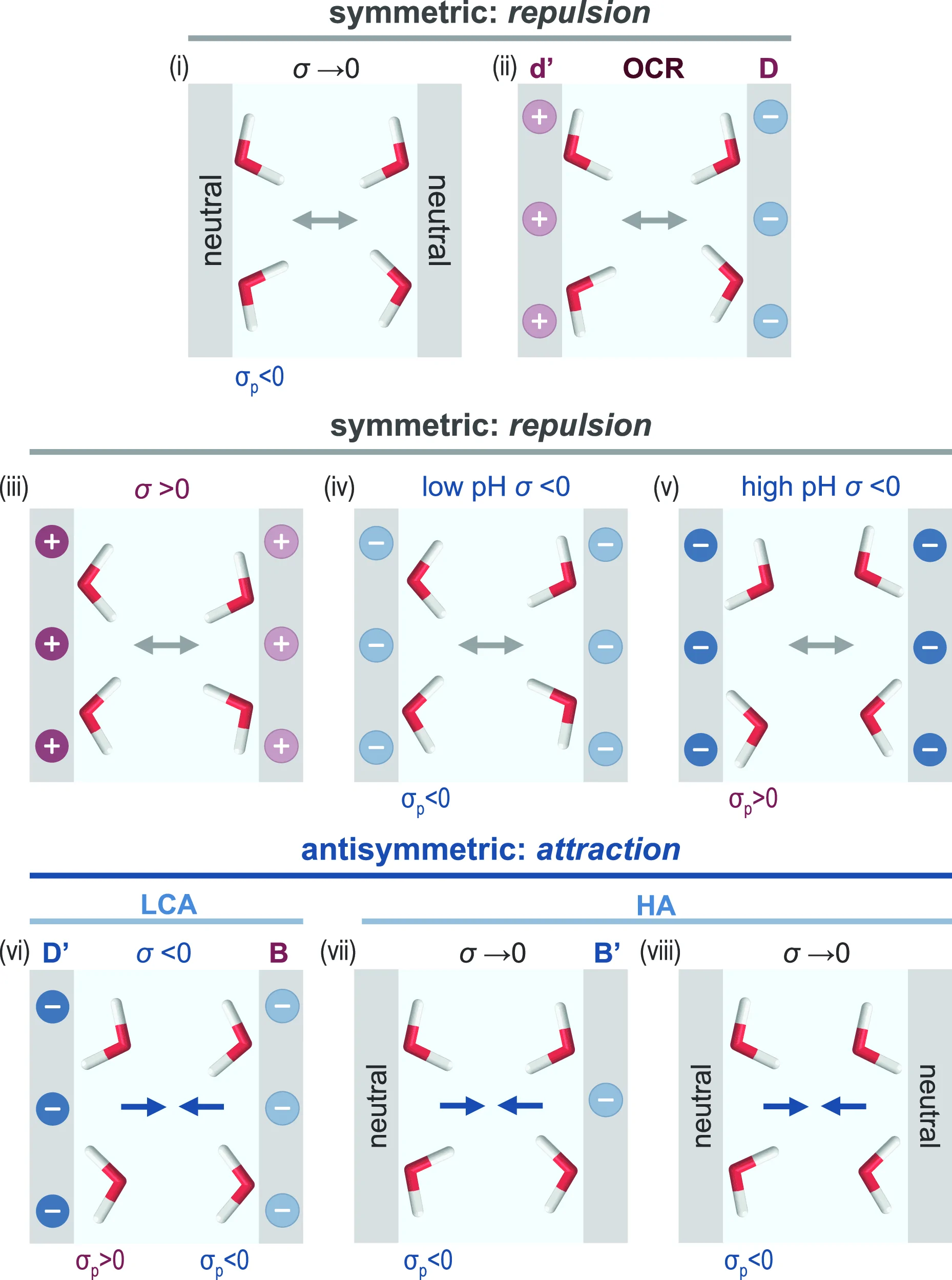
Summary of interaction scenarios and outcomes in water within the electrosolvation view: like-charge attraction (LCA), hydrophobic attraction (HA) and opposite charge repulsion (OCR). *Symmetric interactions (repulsion)*: (i) Formally charge-neutral surfaces may repel solely on account of the electrical effects of interfacial polarization. (ii and iv) Weakly charged surfaces, of like or opposite sign, may repel on account of a sufficiently strong interfacial polarization that dominates the surface charge (OCR: e.g., points *d'* and *D* in Figure 10). (iii and v) The electrical contribution from interfacial polarization reinforces that of the surface charge causing repulsion. Note that scenarios (iv) and (v) could reflect interactions between anionic surfaces at low and high pH respectively. *Antisymmetric interactions (attraction)*: (vi) Like-charged surfaces with dissimilar interfacial polarization (not necessarily opposite in sign) may appear oppositely charged in the far-field producing an attraction at large separations. (vii and viii) Possible scenarios underpinning long-ranged hydrophobic attraction (HA): either a small imbalance in charge (left), or polarizations of opposite sign (right), on two predominantly neutral surfaces could be sufficient to generate an attraction.

Importantly, such an antisymmetric attractive interaction at larger separations does not necessarily imply monotonic attraction to contact (Figure 10C).^45^ For example even within the PB description, oppositely charged particles carrying nonidentical amounts of charge may attract at long-range but repel upon closer approach, at separations of approximately a Debye length.^101^ Physically, the interaction at small separations is governed by entropic repulsion due to the counterions that must remain in the gap in order to satisfy charge neutrality. It is therefore not surprising that a shorter-range repulsion is recovered in interaction free energies calculated using the nonlocal electrostatics framework. Thus, pairs of particles, or even just facing surfaces of objects, whose charge and polarization properties give rise to far-field potentials that lie on opposite sides of the zero-potential contour line in the plot in Figure 10B can give rise to nonmonotonic spatial free energy profiles which are attractive only at long-range, as seen in experiment.

In summary, Figure 10B shows that for interactions in water, this framework immediately yields the possibility of attractive forces for negatively charged particles (quadrant III) and repulsion for positively charged particles (quadrant IV). Experiments show that the scenario can invert in nonaqueous media, yielding attraction for positively charged particles and repulsion between negative particles. Within the present model, such a sign-inversion of the effect would require a change of sign of an interfacial solvent property – e.g., polarization (upper half-plane, quadrants I and II in Figure 10B). In fact an inversion in sign of σ_p_ is expected from MD simulations of alcohols at neutral or weakly charged interfaces. Thus, the nonlocal electrostatics model captures attraction between anionic particles in water and cationic particles in nonaqueous media, and the canonically expected repulsion otherwise (Figure 11).

Furthermore, this theoretical view can in fact capture qualitative trends in the frequently observed p*H* dependence of the attraction (Figure 11, panels iv and v). Here the role of p*H* would mainly seem to be to alter the balance between charge and the interfacial polarization on the particles in solution. To the first order, p*H* simply defines these values and their relative magnitudes at infinite separation. Thus, for anionic particles in water, at low and high p*H*, we may expect either polarization or charge to dominate the far-field interaction, respectively. This would create a situation where the properties of both interacting particles correspond to potential values on the same side of the zero-potential contour in Figure 10B. Thus, interactions between particles would be predominantly “symmetric” and therefore repulsive. At intermediate p*H*, the balance between charge and polarization could be such that particles appear “oppositely charged” to each other, in line with an “antisymmetric” interaction, and long-range attraction ensues (Figure 11, case vi). Thus, while charge regulation upon mutual approach of particles may in practice contribute an additional free energy that is favorable to the attraction, within the nonlocal electrostatics model charge regulation is not a prerequisite for attraction.^4^ Further, antisymmetric interactions between two near neutral, yet slightly negatively charged surfaces in water, may result in attraction which may play a role in the long-range hydrophobic interaction (Figure 11, cases vii and viii).^37,40^ Importantly, the model also envisages *opposite charge repulsion* (OCR) (Figure 11, case ii). Here, particles carrying opposite signs of electrical charge repel rather than attract. This is illustrated by an example pair of particles characterized by properties (d', D) in Figure 10B where the contribution from interfacial polarization in the case of the negative surface dominates the sign of its electrical potential. This places far-field electrical potentials for both surfaces on the same side of the zero-potential contour, and long-range repulsion ensues.

A nonlocal electrostatics model provides deeper insight into the mechanisms driving particle interactions, and clustering in particular, in liquids, successfully overcoming the limitations of the previous, more simplified framework.^4,44,45^ If, pending direct evidence, we were to accept the concept of “antisymmetric interactions” driving attraction between like-charged particles, the crucial experimental feature that would remain to be explained is the surprisingly long range of the putative solvent contribution to the interparticle interaction at very low salt concentrations. We remark here parenthetically, that at higher salt concentrations typical of biologically relevant conditions, modest correlation lengths ξ < 10 Å could be sufficient to explain the counterintuitive and p*H*-dependent cluster formation of highly negatively charged proteins.^102^ However, the long decay lengths of the electrosolvation interaction, now evident in a host of low-salt concentration (*c* < 1 mM) experiments on microspheres, arguably presents the most significant remaining challenge to future models (Figures 3, 8).^9^ Thus, while the present nonlocal electrostatics model does not capture experiments in full quantitative detail, it provides a qualitative framework to guide experimental design and thinking as well as future modeling efforts.

## Exploring Alternate Explanations for the Experimental Observations

It has been long recognized that externally imposed gradients of various kinds – e.g., ions, molecular solutes, and temperature – can cause motion of a fluid at an interface, which can in turn exert forces on suspended particles.^103,104^ It is worth considering carefully whether the broad family of experiments displaying electrosolvation effects may in fact be governed by forces of a different nature, with contributions from gradient-generated forces in particular. A detailed discussion of a possible role for these effects appears in the Supporting Information.

## A Link to Electrochemical Phenomena and Observables

Although it is beyond the scope of the present review to discuss precise connections between the role played by electrosolvation effects in charged-particle interactions and in electrochemical phenomena, we briefly discuss possible links that may provide a focus for future work. At a fundamental level, electrochemical processes occur at interfaces, and the energetics of ions, electrons, and reactants near an electrode are strongly influenced by the local solvent environment. There are however fundamental conceptual challenges associated with measuring the electrical potential due to solvent structuring at an interface. This potential may be referred to as the “*χ* potential” in the electrochemical literature and is related to the quantity termed φ_0_ which denoted the *excess* electrical potential due to the structuring of the interfacial solvent in our original theoretical model of electrosolvation forces^4,77^ (Figure 1B). Although nonlinear spectroscopy methods can probe solvent orientation at an interface, converting measurements of solvent orientation into electrical potentials is model dependent.^74,105^ It turns out that determining the electrical potential due to the solvent from molecular simulations is itself fraught with conceptual difficulties.^106^ The work reviewed here suggests that the properties of interfacial solvent organization can be reflected in long-ranged interaction signatures. Therefore, experimental readouts on interactions may, in conjunction with an appropriate theoretical model, contribute to inferences of the sign and magnitude of the solvent contribution to the electrical potential at an interface. Furthermore, the observations on particle interactions demonstrate that the range and rate of decay of electrical forces are altered by the properties of the solvent. The rate of decay of the force between two particles is intimately connected with the extent of the diffuse part of the electrical double layer. We have shown that within a nonlocal electrostatics model, solvent-governed and ion-governed length scales can couple together to give an effective screening length of the form of eq 3 that is different from the traditional Debye length.^44,45^ Many measurable electrochemical and electrokinetic observables—including double-layer capacitance, differential capacitance, zeta potential, and electro-osmotic mobility—depend sensitively on the spatial structure and extent of the interfacial region, and are therefore characterized and influenced by the screening length. For example, electrokinetic mobilities may depend on the polarization charge at the particle-solvent interface and the influence thereof on the diffuse double layer. Indeed, there is growing recognition that interfacial solvent organization may contribute significantly to measurable electrokinetic properties, although the sign, magnitude and precise nature of these contributions remain the subject of active investigation.^107–112^ Thus, several phenomena of interest in electrochemistry involve interfacial solvent structure and polarization effects that are conceptually related to the mechanisms underlying the fluid-phase interactions discussed here. Although direct links between interaction measurements and specific electrochemical observables remain insufficiently established at present, exploring these connections in future work may help bridge these currently rather separate perspectives on interfacial phenomena and advance a more unified physical picture.

## Future Directions and Open Frontiers

Understanding interactions in the relatively simple situation of a pair of particles immersed in a fluid holds fundamental significance. It is becoming increasingly clear that the traditional view of interactions fails to qualitatively capture the observed behavior in a wide range of experimental situations, especially at long range. Alongside a number of related studies, the work reviewed here makes the experimental case for a revision and upgrade of standing models of interparticle interactions. These developments will of course need to run in tandem and in close association with independent experimental investigations, e.g., using spectroscopy, as well as with molecular simulations of fluid properties in the vicinity of nanoscopic and microscopic objects.

Setting aside the central question on the origins of the micron-scale range of the electrosolvation force between microspheres (Figure 8), the full picture on exactly how interfacial solvation may influence far-field interactions, and specifically, the role played by charge asymmetry in this scheme is likely to see much further development in the coming years. For example, in contrast to atomic ions, molecular anionic and cationic groups display differences in solvation that are influenced by steric considerations and bonding interactions of the ionizable groups. The properties of the interfacial solvent in these cases may not be solely governed by the object’s charge. Thus, the most general underlying principle could lie along the lines of “differential solvation” of the interfacial groups, a significant aspect of which could involve charge-governed effects. The experimental observations on interactions in EG, as well as interfacial water-structure measurements at lipid-water interfaces, furnish instructive examples (Figure 3).^69–71^

Furthermore, it is possible that the role of p*H* in the electrosolvation attraction is more nuanced than previously held.^4^ In the original model of the interaction, charge regulation upon approach of the particles was necessary for the attraction to occur. But experiments involving particle coatings composed of highly acidic polyelectrolytes such as PSS, as well as DNA and LBs composed of phosphate groups have shed more light on this picture^7,9^ (Figure 7). The trends observed to date raise the possibility that for a number of materials – especially those carrying a high density of the strongly anionic phosphate and sulfonate groups – the charge/molecular-solvent-property (polarization) relationship may be inherently situated in a regime that fosters attraction. While charge regulation could occur and indeed contribute to the overall interaction depending on the specific experimental situation, it may not play an essential role in generating an attraction.

Next, the ramifications of the electrosolvation interaction at smaller, submicrometer length-scales could be rather broad, potentially impacting understanding in areas ranging from structural biology to cellular spatial organization, as well as in origin-of-life and in disease research. For example, we may expect major shifts in our view of the principles governing biomolecular organization, e.g., in problems such as the packing of DNA in chromatin, and protein and nucleic acid folding. Experimental evidence that the range of the interaction between charged particles in solution can be much longer than previously held, as well as the experimental demonstration that DNA can sustain a long-range attraction, could have important implications for chromatin organization.^9^ The electrical charge on DNA is manifestly negative and far from completely canceled by histones.^113^ Yet chromatin condenses — presumably facilitated by the rapid spatial screening of the canonical repulsion at physiological salt concentrations. It may be worth reconsidering the chromatin condensation problem in light of the evidence that rather than repelling strongly, DNA may in fact sustain self-attraction.^9,114,115^ A natural consequence of this insight could point to a general role for attractive long-ranged electrosolvation forces in the compaction of chromatin and more generally in the condensation of charged matter in biology.^116–118^

Finally, long-range electrical effects founded in the nonlocal properties of water may well play a key role in driving biomolecular folding. This is because, within the nonlocal electrostatics view, built-in structural dipoles and quadrupoles, and possibly even higher-order multipoles, may all be expected to produce long-range electrical signatures. One might reasonably entertain the idea that the protein folding process is influenced, and possibly even directed, by intrinsic electrical features of molecular structure – supplying an ingredient to the resolution of Levinthal’s paradox.^119,120^ To conclude is to quote one final time from Barry Ninham’s seminal review: “. . .we can expect that our coming to grips with the solvent over the coming years will be revolutionary in colloid science, especially as it pertains to biology”.^3^

## Supplementary Material



## Notes

Additional discussion exploring alternate explanations for the experimental observations (PDF)
