# Supplementary material for: The Electrosolvation Effect: The Medium Is the Message
Source: Langmuir. 2026 Sep 9;42(40):29602–21. doi: 10.1021/acs.langmuir.6c02385 (PMC13674290; doi:10.1021/acs.langmuir.6c02385)
Supplement: Supplementary file 1 [file la6c02385_si_001.pdf]

The electrosolvation effect: the medium is the message

(Supporting Information)

Sida Wang[1], Ali Behjatian[1], Angela Le[1], Xin Zhu[1], Madhavi Krishnan*[1,2]

[1]Physical and Theoretical Chemistry Laboratory, Department of Chemistry, University of Oxford, South Parks Road, Oxford OX1 3QZ, United Kingdom

[2]The Kavli Institute for Nanoscience Discovery, Sherrington Road, Oxford OX1 3QU, United Kingdom.

*correspondence to madhavi.krishnan@chem.ox.ac.uk

Exploring alternate explanations for the experimental observations

It has been long recognised that externally imposed gradients of various kinds – e.g., ions, molecular solutes, and temperature – can cause motion of a fluid at in interface, which can in turn exert forces on suspended particles[1, 2]. It is worth considering carefully whether the broad family of experiments displaying electrosolvation effects may in fact be governed by forces of a different nature, with contributions from gradient-generated forces in particular.

The experiments are performed at constant temperature, with low intensity and spatially uniform optical illumination, and with no known externally imposed gradients – chemical or physical. In order to promote ionization of the surface groups anionic particles are often rinsed in sodium hydroxide prior to extensive centrifugation, rinsing and interaction measurements. However we have also performed experiments on silica particles without prior treatment with a base. Here particles are rinsed multiple times, incubated for 12 hours in deionized water, then rinsed thoroughly again prior to measurement. Particles display the characteristic cluster formation behaviour, effectively eliminating the possibility of externally imposed ions gradients in driving interactions[3]. Regardless, it is worth examining the possibility, however improbable, that particles resuspended in a different medium may either leach or attract species such as salt ions or small molecules into or from bulk solution, thus acting as sources or sinks, radiating chemical gradients into the medium to which neighbouring particles respond. Forces arising from such gradients would in fact be rather long ranged, and are therefore worth carefully exploring for their possible impact on interparticle interactions.

We assume for the sake of argument that the concentration gradient around a particle is set up by diffusion of a symmetric z:z salt outwards from the particle. The spatial profile of such a gradient may therefore be given by the solution to the diffusion equation:

$$c \propto \exp(-r^2/\sigma^2) \quad \text{(S1)}$$

The velocity of a charged particle of radius R exposed to a gradient in salt concentration c has both diffusiophoretic and chemophoretic components. We may use this velocity to estimate the total force on a test particle exposed to such a gradient and examine its sign under various conditions. In one dimension this force is given by

$$\boldsymbol{F}_{\mathrm{grad}} \cong \frac{3k_{\mathrm{B}}T}{2z^2}\frac{R}{l_{\mathrm{B}}}\left(\beta\tilde{\zeta} + \frac{\tilde{\zeta}^2}{8}\right)\frac{\mathrm{d}\ln c}{\mathrm{d}r} \qquad \text{(S2)}$$

Here $\beta = \frac{D^- - D^+}{D^- + D^+}$ is a function of the diffusivities of anions and cations D^- and D^+ respectively, and $\tilde{\zeta} = \frac{e\zeta}{k_{\mathrm{B}}T}$ is the dimensionless zeta potential of the particle. $l_{\mathrm{B}} = \frac{e^2}{4\pi\varepsilon\varepsilon_0 k_{\mathrm{B}}T}$ is the Bjerrum length in a medium of dielectric constant ε and is about 0.7 nm in water.

A qualitative analysis of the force on our test particle in the vicinity of a “source particle” shows that a diffusiophoretic force does in fact contain the charge asymmetric feature seen in the experiments. Assuming particles act as sources of a salt AX, where e.g., A^+ ions diffuse more slowly than their anionic counterpart X^-, we have $\beta < 0$. This condition is met for salts like NaCl and NaOH in water. Since $\frac{\mathrm{d}\ln c}{\mathrm{d}r} < 0$, negative particles characterized by $\zeta < 0$ would move up the salt gradient due to a neighbouring particle, implying interparticle attraction; positively charged particles ($\zeta > 0$) would move down the gradient and therefore appear to repel each other. In an alcohol, we might expect weaker solvation of the ions to invert the ordering of diffusion coefficients of the cations and anions such that $D^+ > D^-$. Now positive particles would move up the concentration gradient implying an observed attraction for positives and repulsion for negative particles, as seen in experiments. Thus the diffusiophoretic component of the total force (first term in Eq. (S2) in a salt concentration gradient does contain the crucial charge-symmetry breaking feature observed in experiments. However, the second, chemophoretic contribution is agnostic to

the sign of charge of the particle and implies a force that is always attractive, albeit generally weaker than its diffusiophoretic counterpart.

Leaving aside an estimate of the magnitude of the force for the moment, we note that although diffusiophoresis carries the key charge-asymmetric feature of the experimental observations, modulation of an interparticle attraction due to diffusiophoresis in the range of ways demonstrated in the experiments would be much harder to explain. Consider the experimentally observed pH dependence of the attraction both in water and alcohols. In experiment, high (low) pH abolishes the attraction for negative (positive) particles in water (alcohols). A diffusiophoretic mechanism would in fact point to an enhancement of the attraction under such conditions due to proportionality of the force to both $\beta\zeta$ (diffusiophoretic contribution) and ζ^2 (chemophoretic term). Moreover the ability to modulate and indeed totally abolish interparticle attraction in water through the addition of trace quantities of surface active agents speaks strongly for an interfacially-governed mechanism; it is hard to envisage a scenario where putative concentration gradients in ions around particles are totally eliminated by the presence of micromolar or millimolar concentrations of amino acids or surfactants. It would also be entirely unclear how attraction-promoting ion gradients could continue to persist in the presence of diffusion barriers such as multiple layers of polyelectrolytes and lipid bilayers coating the microspheres. Further, the observation of charge asymmetric attraction in water-alcohol mixtures would also be difficult to reconcile with a diffusiophoretic mechanism. A concentration gradient in a neutral solute (in this case the solvent at lower concentration) decaying outwards from a particle would produce forces that act on particles pushing them apart regardless of their charge. Explaining the charge dependent symmetry-breaking attraction observed in water-alcohol mixtures using a diffusiophoretic mechanism would therefore require particles to always act as sinks of whichever solvent is present

at lower concentration, irrespective of whether it is water or alcohol. It seems implausible that such conditions would be met rather broadly in practice.

To quantitatively assess the role of gradient-induced forces, we may now estimate the magnitude of a putative attractive diffusiophoretic force on a negatively charged particle with $\tilde{\zeta} \sim -1$ in water, at an intersurface separation $x \approx 1$ μm from a source particle, about two minutes after salt ions have begun leaching out of the particles into the medium. Note that $-1 < \beta < 1$ with typical values around -0.19 for NaCl and -0.007 for KCl. Here we assume a rather large disparity between the diffusion coefficients of the cation $D^{+} = 10^{-9}$m^2/s and the anion $D^{-} = 5 \times 10^{-9}$m^2/s which gives $\beta = -0.67$. In Eq. (S1) we have $\sigma^2 = 4Dt$, where $D = 10^{-9}$ m^2/s represents a nominal diffusion coefficient for the ions. We estimate the force at $t = 100$ s which represents the time elapsed between the start of the experimental observation after the final rinsing and resuspension of the particles in the measurement medium. Longer observation times t would imply proportionately weaker forces at the same interparticle separation. The estimated force on a 5 μm diameter particle is of the order of 100 aN, which is more than 3 orders of magnitude smaller than the attractive forces of magnitude ca. 10-100 fN and larger measured in experiment[4] (Fig. 8B). Moreover, the linear dependence of the diffusiophoretic force on x would suggest a form for the attractive part of an effective potential of mean force that grows quadratically with separation. This is markedly different from the exponential decay measured in experiments[4] (see Fig. 8B). All these factors suggest that a solvent-influenced diffusiophoretic mechanism is highly unlikely to account for the large body of experimental evidence on the observed interaction.

References

(1) Anderson, J. Colloid transport by interfacial forces. *Annual Review Of Fluid Mechanics* **1989**, *21*, 61–99.

(2) Paustian, J.; Angulo, C.; Nery-Azevedo, R.; Shi, N.; Abdel-Fattah, A.; Squires, T. Direct Measurements of Colloidal Solvophoresis under Imposed Solvent and Solute Gradients. *Langmuir* **2015**, *31* (15), 4402–4410. DOI: 10.1021/acs.langmuir.5b00300.

(3) Wang, S.; Walker-Gibbons, R.; Watkins, B.; Flynn, M.; Krishnan, M. A charge-dependent long-ranged force drives tailored assembly of matter in solution. *Nature Nanotechnology* **2024**, *19*, 485–493. DOI: 10.1038/s41565-024-01621-5.

(4) Wang, S.; Le, A.; Walker-Gibbons, R.; Krishnan, M. Direct measurement of the attractive electrosolvation force between a pair of colloidal particles. *Reports on Progress in Physics* **2026**, *89* (2), 028101. DOI: 10.1088/1361-6633/ae3fca.
